# Abortion stigma amongst the public in high-income countries: a mixed-method systematic review

**DOI:** 10.1080/26410397.2026.2622203

**Published:** 2026-02-09

**Authors:** Jana Niemann, Marie Bernard, Dennis Jepsen, Nadja Freymüller, Laura Weinhold, Céline Miani, Claudia Luck-Sikorski

**Affiliations:** a Research Associate, Institute of Medical Sociology (IMS), Medical Faculty, Martin Luther University Halle-Wittenberg, Halle (Saale), Germany; d Research Associate, Department of Sustainable Environmental Health Sciences, Medical School OWL, Bielefeld, Germany; Research Associate, Institute for Rehabilitation Medicine, Medical Faculty, Bielefeld University, Martin Luther University Halle-Wittenberg, Bielefeld, Germany; e Research Assistant, Institute of Medical Sociology (IMS), Medical Faculty, Martin Luther University Halle-Wittenberg, Halle (Saale), Germany; f Professor of Social and Gender Epidemiology, Department of Epidemiology and International Public Health, School of Public Health, Bielefeld University, Bielefeld, Germany; g Professor of Mental Health and Psychotherapy, SRH University of Applied Sciences Heidelberg, Campus Gera, Gera, Germany

**Keywords:** abortion, pregnancy termination, abortion stigma, social stigma, stigma, social discrimination, Reproductive Health, public attitudes

## Abstract

The public plays a central role in producing and sustaining abortion stigma by shaping dominant narratives, reinforcing moral norms, and passing judgment on individuals associated with abortion. These collective attitudes are expressed through social exclusion and symbolic condemnation – practices that shape both personal experiences and structural access to care. This mixed-methods systematic review updates the state of research by synthesising recent evidence from high-income countries (HICs), with particular focus on how the public enact and experience abortion stigma. We conducted a mixed-methods systematic review of peer-reviewed quantitative and qualitative studies published since 2015, following international standards for systematic reviews. Due to heterogeneity in measurement, quantitative and qualitative data were narratively synthesised. Methodological quality was assessed using standardised appraisal tools for both quantitative and qualitative research. Nineteen studies were included (12 qualitative, 7 quantitative). Quantitative findings reveal that abortion stigma in HICs persists at moderate levels and is associated with religiosity, political conservatism, lower income, and male gender. Qualitative studies demonstrate how stigma is enacted, perceived, and anticipated across diverse social settings, highlighting prevailing stereotypes and uncovering experiences of verbal harassment and social exclusion. Abortion stigma remains deeply embedded within the public. By updating and expanding on the previous work, this review underscores the need for targeted, group-specific stigma reduction strategies and more robust instruments for capturing stigma.

## Plain language summary

This review looked at research on how people in wealthy countries view abortion stigma. Abortion stigma means people who have abortions or those who support or provide them face judgment, shame, or exclusion. Public opinion is shaped by debates, the media, and activism. We reviewed 19 studies from high-income countries. These used surveys, interviews, experiments, and media analysis. The studies showed that people’s attitudes toward abortion vary a lot: some disapprove strongly, while others are supportive. Stigma often comes from beliefs about gender, sexuality, and morality. For example, people seeking abortions may be seen as irresponsible or morally wrong, especially if they do not fit the traditional ideas of womanhood. Stigma is not just about personal opinions. It is also shaped by social norms, politics, and the media. Even abortion rights advocates may face stigma. Most studies we found were from the United States so more research is needed in other wealthy countries to get a fuller picture. Overall, abortion stigma remains common in public life. Reducing this will require ongoing, culturally sensitive efforts that encourage respectful and supportive conversations about abortion.

## Résumé

Le public joue un rôle central dans la production et le maintien de la stigmatisation de l’avortement en façonnant les discours dominants, en renforçant les normes morales et en portant un jugement sur les personnes associées à l’avortement. Ces attitudes collectives s’expriment par l’exclusion sociale et la condamnation symbolique, pratiques qui influent aussi bien sur les expériences personnelles que sur l’accès structurel aux soins. Cette revue systématique à méthodes mixtes actualise l’état de la recherche en synthétisant des données récentes provenant des pays à revenu élevé, en se centrant particulièrement sur la manière dont le public édicte et vit la stigmatisation de l’avortement. Nous avons mené une revue systématique à méthodes mixtes d’études quantitatives et qualitatives évaluées par les pairs et publiées depuis 2015, conformément aux normes internationales pour les revues systématiques. En raison de l’hétérogénéité des mesures, les données quantitatives et qualitatives ont été synthétisées de manière narrative. La qualité méthodologique a été évaluée au moyen d’outils d’appréciation normalisés des recherches qualitatives et quantitatives. Dix-neuf études ont été incluses (12 qualitatives, 7 quantitatives). Les résultats quantitatifs révèlent que la stigmatisation de l’avortement dans les pays à revenu élevé persiste à des niveaux modérés et qu’elle est associée à la religiosité, au conservatisme politique, à un faible revenu et au genre masculin. Les études qualitatives démontrent comment la stigmatisation est édictée, perçue et anticipée dans divers contextes sociaux, mettant en lumière les stéréotypes dominants et dévoilant des expériences de harcèlement verbal et d’exclusion sociale. La stigmatisation de l’avortement demeure profondément ancrée dans la société. En actualisation et en approfondissant les précédents travaux, cette revue souligne la nécessité de disposer de stratégies ciblées et adaptées aux groupes pour réduire la stigmatisation ainsi que d’instruments plus performants pour la mesurer.

## Resumen

El público desempeña un papel central en producir y sostener el estigma del aborto al moldear las narrativas dominantes, reforzar las normas morales y juzgar a personas asociadas con el aborto. Estas actitudes colectivas son expresadas por medio de la exclusión social y la condena simbólica: prácticas que influyen en las experiencias personales y en el acceso estructural a los servicios. Esta revisión sistemática de métodos mixtos actualiza el estado de las investigaciones al sintetizar evidencias recientes de países de altos ingresos (PAI), con un enfoque específico en cómo el público ejerce y experimenta el estigma del aborto. Realizamos una revisión sistemática de métodos mixtos de estudios cuantitativos y cualitativos revisados por pares publicados desde 2015, siguiendo las normas internacionales de revisiones sistemáticas. Debido a la heterogeneidad en la medición, los datos cuantitativos y cualitativos fueron sintetizados de manera narrativa. La calidad metodológica fue evaluada utilizando herramientas de evaluación normalizada tanto para las investigaciones cuantitativas como para las cualitativas. Se incluyeron 19 estudios (12 cualitativos, 7 cuantitativos). Los hallazgos cuantitativos revelan que el estigma del aborto en los PAI persiste a niveles moderados y está asociado con religiosidad, conservadurismo político, menores ingresos y género masculino. Los estudios cualitativos demuestran cómo se ejerce, percibe y prevé el estigma en diferentes entornos sociales, lo que destaca los estereotipos predominantes y revela experiencias de acoso verbal y exclusión social. El estigma del aborto sigue profundamente arraigado en el público. Al actualizar y ampliar el trabajo anterior, esta revisión subraya la necesidad de formular estrategias de reducción del estigma focalizadas en grupos específicos y crear instrumentos más robustos para capturar el estigma.

## Introduction

Abortion services are essential components of healthcare and a fundamental human right, grounded in international human rights law and global health frameworks.^1–3^ However, universal access to safe abortion services remains uneven and inconsistent across contexts.^1,4^ One significant barrier is the persistent stigma surrounding abortion, which is deeply rooted in the dominant cultural notions of womanhood that reduce female sexuality to a solely reproductive function.^5^ This stigma plays a central role in preventing abortions from being recognised and implemented as a universally accessible human right, by constraining the ability to make free and informed decisions about one's own body.^6^

Stigma operates on multiple, intersecting levels. At the individual/micro level, people who seek, provide, or support abortion services are often subjected to stereotyping, marginalisation, and discrimination.^5,7,8^ At the institutional/meso level, abortion stigma manifests in practices such as the separation of abortion services from other healthcare procedures and insufficient training of healthcare professionals.^5^ At the structural/macro level, stigma is reflected in restrictive laws and policies that limit or prohibit access to abortion care.^5^ Such legal and policy restrictions are not neutral; they represent systemic violations of the right to health^9,10^ and the right to bodily autonomy, as recognised by international human rights bodies.^3,11,12^

These levels of stigma are not isolated but mutually reinforcing. Structural stigma, such as the persistent debate surrounding the (de)criminalisation and legalisation of abortion,^13–16^ shapes broader societal attitudes.^8^ Public opinion influences how this debate is framed and sustained, creating a self-perpetuating cycle.^8^ This dynamic is maintained through collective beliefs, social interactions, and dominant cultural narratives that normalise stigma and entrench barriers to care.^17–19^ As a result, stigma continues to shape both individual perceptions and institutional practices, manifesting as social exclusion, moral judgment, and implicit biases that inhibit open discourse and hinder access to abortion services.^8^

Thus the public plays a crucial role in perpetuating abortion stigma and shaping the social attitudes and norms that circulate within communities, neighbourhoods, and broader social networks. To break this vicious cycle and to establish abortion as both a fundamental human right and an essential healthcare procedure (legally, socially, institutionally), it is necessary to assess current public opinion toward abortion and the extent to which it remains stigmatised. Understanding these dynamics is key to informing policies and public discourses that promote equitable and stigma-free access to abortion care.

### Stigma in the crowd: who we mean when we say “the public”

Public stigma refers to the widespread negative attitudes and judgments present within society and among influential social actors, which contribute to and sustain the stigmatisation of individuals with health-related conditions.^20^ Yet, it is important to clarify whom we refer to when talking about “the public”. The term “public” typically refers to a broader population that may not have direct personal experience with abortion care. However, this category also includes more visible and organised actors, such as pro-choice and anti-abortion activists, journalists, and opinion leaders, who actively shape discourse and influence the societal framing of abortion. To reflect this distinction, in this review, we use the term “public” to refer to both the general population and these engaged subgroups, while the term “general public” is reserved for the broader, undifferentiated population.

Public members can enact, perceive, experience, and anticipate stigma in different ways. Enacted stigma refers to overt expressions of stigmatisation, such as discriminatory behaviour, stigmatising language, or support of structural barriers to abortion access.^21^ In contrast, perceived stigma describes individuals’ awareness of negative attitudes toward abortion, whether they personally hold these views or believe them to be widespread. This overlaps with the concept of public stigma, which focuses on collective reactions toward individuals associated with a stigmatised issue, such as abortion.^22,23^

While some members of the public enact stigma through judgmental attitudes or exclusionary behaviour,^7^ others, particularly abortion rights advocates, may themselves become targets of stigmatisation.^24^ These groups might experience backlash, moral condemnation, or social marginalisation. In this context, they may also anticipate stigma, which describes the expectation of being judged or excluded by their public association with abortion. Importantly, this dynamic illustrates how abortion stigma not only restricts healthcare access^25^ but also curtails freedom of expression, association, and participation in public debates^8^ – rights that are central to democratic societies.

### Review context, objectives and research questions

In 2016, Hanschmidt et al^7^ conducted a systematic review identifying three quantitative and two qualitative studies on public abortion stigma. Despite a heterogeneous focus and diverse measurements and definitions of abortion stigma, these studies found stigmatising attitudes toward women who have abortions, including considering them less desirable, “inexcusable”, or even classifying them as murderers.^7^ Given the expanding body of research on abortion-related stigma among the public, updating the systematic review by Hanschmidt et al^7^ is both timely and necessary.

#### Objectives

The present review aims to critically examine abortion-related stigma among the public in high-income countries (HICs) using a mixed-methods systematic approach. To address this, we begin by mapping how recent studies define and conceptualise abortion stigma, including whether definitions extend beyond individual attributes to broader social power dynamics. This is important because divergent definitions, from individual traits to broader social power dynamics, shape research priorities, interventions, and whether abortion is framed as a healthcare service or a human right. Thus this review addresses Millar’s critique^26^ that prior research often relies on narrow individual-level definitions. In addition, we will examine the extent, forms, and factors associated with public abortion stigma from a quantitative perspective. Finally, we will synthesise qualitative evidence on lived experiences and perceptions of stigma in HICs.

#### Why does this review focus on high-income countries

As detailed in our study protocol,^27^ notable disparities exist in legal abortion access between HICs and low- and middle-income countries.^28^ Individuals in HICs generally benefit from broader access to comprehensive sexual and reproductive health care, including abortion services.^29^ Conversely, unsafe abortions are more common in low- and middle-income countries and can strongly shape public perceptions of abortion. This review focuses exclusively on HICs to ensure consistent and comparable insights.

#### Research questions

We thereby aimed to answer the following research questions:

**RQ1**: What definition(s) did the studies employ for their theoretical conceptualisation of abortion-related stigma?

**RQ2**: To what extent, and in what forms, does abortion-related stigma manifest among the public from a quantitative perspective?

**RQ3**: What factors are associated with abortion-related stigma from a quantitative perspective?

**RQ4**: What are the lived experiences concerning abortion stigma among the public in HICs from a qualitative perspective?

Building on our broader research efforts, this review is the third and final part of a systematic review series examining abortion-related stigma from different perspectives. In addition to the present review, which focuses on the public, we conducted two separate reviews addressing the perspectives of individuals who have abortions and abortion providers. While an earlier review by Hanschmidt et al^7^ considered all three perspectives in a combined analysis, our approach was to examine each viewpoint in greater depth through separate, focused reviews. This allowed for a more detailed understanding of how stigma is experienced and expressed across distinct groups.

## Methods

This systematic review was carried out following the PRISMA guidelines^30^ and the Joanna Briggs Institute (JBI) guidelines for mixed-methods systematic review (MMSR).^31^ The protocol for this review was published in advance.^27^

### Inclusion criteria

#### Participants

This systematic review included studies of abortion-related stigma among the public.

#### Phenomena of interest

We examined research that investigated the magnitude and manifestation of abortion stigma (prevalence or covariates) among the public.

#### Context

We only included studies from HICs.

#### Types of studies

We selected peer-reviewed studies employing diverse methodologies, namely quantitative, qualitative, and mixed-method designs. Quantitative studies included both clinical and non-clinical studies. The qualitative research utilised comprehensive interviews, focus group discussions, social media content examinations, and autobiographical work.

### Search strategy

From January to February 2023, updated on 27 February 2024, we conducted a thorough review of peer-reviewed literature on abortion stigma in HICs, covering qualitative, quantitative, and mixed-methods research. MEDLINE, CINHAL, PsychINFO (via EBSCOhost), LIVIVO, and Cochrane Library were searched by two researchers (JN and MB) using keywords from a previous review^7^: [abortion OR termination of pregnancy OR voluntary interruption of pregnancy] AND [stigma* OR discrimination*], limiting the search to titles and abstracts. This review builds on that of Hanschmidt et al^7^ and focuses on the studies published after March 2015.

### Study selection

**Figure 1.**
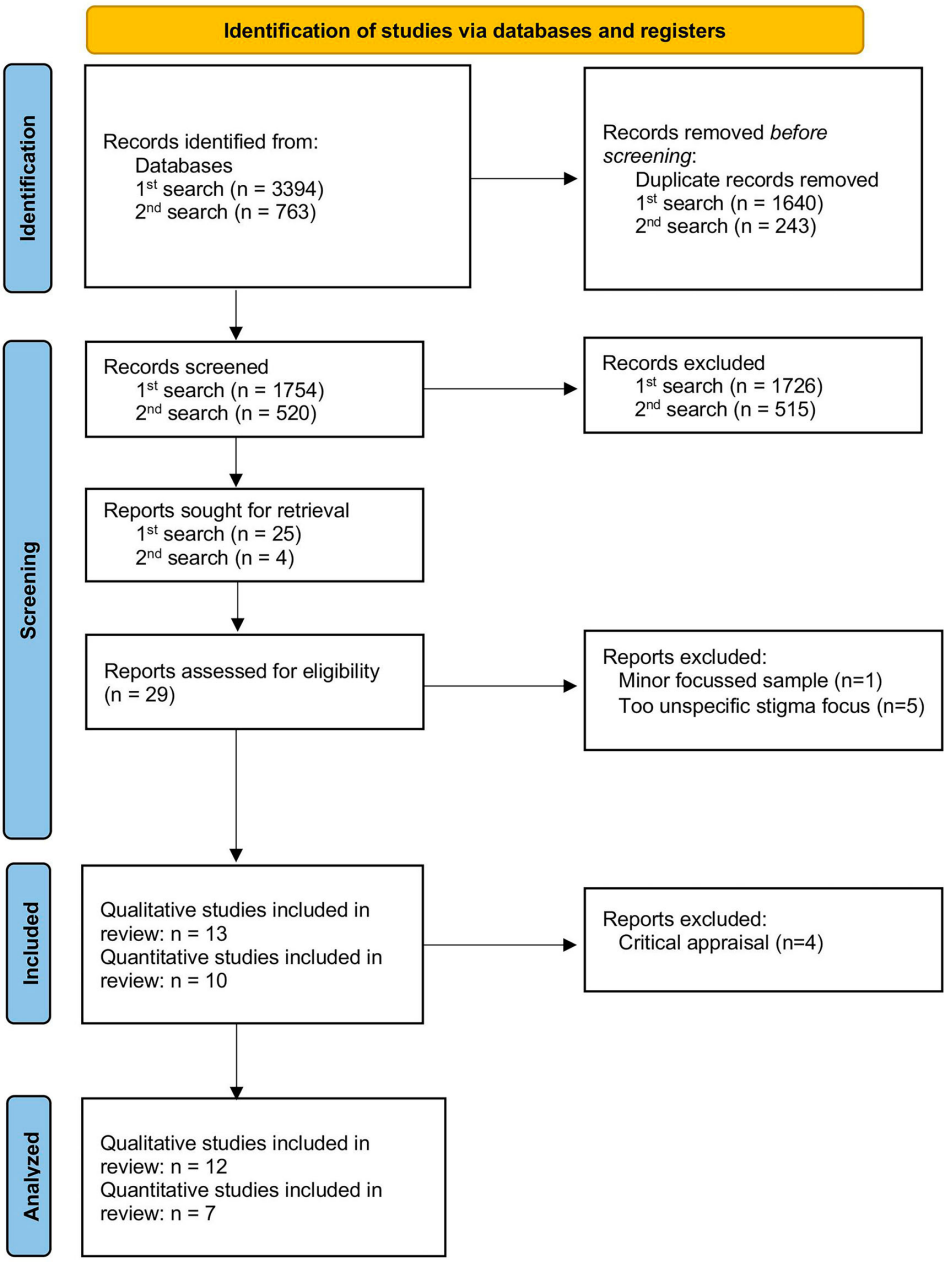
PRISMA Flow Chart. Figure depicts the stages and outcomes of the search and selection process

The identified publications were imported into Rayyan,^32^ and duplicates were removed. Two independent researchers (JN and MB) screened titles and abstracts based on the inclusion criteria. Eligible studies were transferred to Excel for full-text review by JN and MB, while reasons for exclusion were documented. Disagreements regarding the selection were resolved through discussion (Figure 1).

### Critical appraisal

#### Quantitative studies

DJ and JN evaluated the methodological quality and the risk of bias in the included seven quantitative studies using the ROBINS-E tool (Risk Of Bias In Non-randomised Studies – of Exposures).^33^ Any disagreements were resolved through a collaborative discussion.

#### Qualitative studies

JN used the JBI-QARI (Joanna Briggs Institute – Qualitative Assessment and Review Instrument) checklist^34^ to assess the quality of the qualitative studies, as it is a well-established tool specifically designed for interpretive and critical research. Its structured criteria support consistent evaluation of methodological rigour and enhance the transparency and reproducibility of qualitative evidence synthesis. LW reviewed 20% of the studies on thoroughness. The inter-rater reliability, measured by Krippen–Dorff's alpha coefficient,^35^ was 0.945. Disagreements in the evaluations were resolved through discussions until a consensus was reached.

### Data extraction and synthesis

Following the JBI guidelines for MMSR,^31^ we employed a convergent separated approach to independently analyse and integrate quantitative and qualitative data from the studies. MAXQDA (VERBI Software, Berlin, Germany) was used for data extraction and coding in both research strands, as it enables the systematic management, organisation, and analysis of textual data, thereby supporting transparency and methodological rigor in the synthesis process.

#### Quantitative studies

MB extracted information from the articles related to abortion stigma. The analysis encompassed the definition of stigma, study location, participant count, measures of reliability, prevalence statistics, and correlations with other factors. Considering the heterogeneity in measurement approaches across quantitative studies, the team eschewed a meta-analysis in favour of narrative synthesis^31^ executed by MB.

#### Qualitative studies

We followed the JBI meta-aggregation approach for qualitative studies.^34^ During coding, we found that the study populations were too diverse for meta-aggregation. Therefore, JN and MB narratively summarised the content and methodology of the studies based on their findings (cf. Supplementary Table 1). JN coded and extracted the findings of the studies on abortion stigma from the original documents using MAXQDA. She summarised all findings in a table and coded the Table again with NF to identify common themes. Based on this process, MB and NF refined the themes, and MB narratively described them extensively. JN, NF, and MB validated the findings. JN also extracted general information on the definition of stigma, study location, participant count, methodology, and methods of data collection and analysis.

#### Integration of quantitative and qualitative evidence

After analysing the quantitative and qualitative data separately, we used configurative analysis to assess the compatibility of the synthesised findings from both methods. Following the JBI MMSR Methods guidelines,^31^ MB and JN conducted the integration.

### Reflection and reflexivity statement

Our team brings backgrounds in sociology, public health, psychology, social epidemiology, and nutritional sciences. All members were identified as cisgender and white, and we acknowledge that these positionalities shaped the study’s conceptualisation and writing. We approach abortion as a fundamental reproductive right and see stigma as a central barrier to universal healthcare. In this review, we engaged both with the participants’ voices and the interpretations of the study authors.

## Results

Of the 4157 records identified, 24 studies met the inclusion criteria and were critically appraised and summarised in the MMSR.

### Critical appraisal

#### Quantitative studies

In total, we assessed the risk of bias of 10 quantitative studies using the ROBINS-E tool. Four studies were judged to have a low risk of bias,^36–39^ while two studies raised some concerns.^40,41^ One study was classified as having a high risk of bias.^42^ Three studies were excluded from the analysis: one due to an inadequate sample,^43^ and two because of a very high risk of bias arising from uncontrolled confounders.^44,45^.

#### Qualitative studies

Using the JBI critical appraisal tool,^34^ we evaluated most studies as having a moderate level of reliability.

According to item Q8 of the JBI checklist, which assesses whether a study appropriately represents participants’ perspectives, all but one study met this criterion. The study that did not^46^ was excluded from the synthesis due to insufficient representation of participants’ views relevant to our research question. Following this exclusion, a total of 12 studies remained eligible for the qualitative synthesis. Supplementary Table 2 provides additional details regarding the critical assessment.

### Main description

Nineteen studies were selected for the data extraction. Twelve were qualitative and seven were quantitative. Twelve studies were conducted in the United States (US),^36,38–42,47–52^ two each in Australia^18,53^ and Canada^54,55^, and one study was conducted in Germany,^37^ Italy,^56^, and Northern Ireland/Republic of Ireland.^57^ Supplementary Table 3 details the characteristics of all the included studies.

### RQ1: Used definitions for abortion stigma

Across the studies included in this review, the conceptualisation of abortion stigma varied in subject dimensionality and level of analysis, attention to drivers, and engagement with power. Most studies employed at least one explicit definition of abortion stigma,^18,36–42,45,47,48,52,53,55–57^ often referencing multiple sources. Four qualitative studies did not provide an explicit definition of how they define abortion stigma in their respective study^47,49,50,54^ (cf. Supplementary Table 4).

#### Subject dimensionality and level of analysis

##### Individual subject and level

Eleven studies^7,18,36,38,40,42,48,53,55–57^ drew on definitions centred on abortion seekers, particularly those by Kumar, Hessini, and Mitchell,^5^ who view abortion stigma as rooted in cultural norms about womanhood and sexuality. These definitions highlight how abortion is framed as deviant from reproductive expectations, often resulting in shame, marginalisation, or social punishment. Three studies^38,56,57^ applied Cockrill and Nack’s^58^ typology of internalised, felt, and enacted stigma, which focuses on personal and social manifestations of stigma and the attribution of negative traits to individuals who do not conform to normative ideals of femininity.

##### Expanded subjects and multilevels

Ten studies^36–40,42,45,48,55,56^ broaden the subject to include stigma experienced by abortion providers and at the community level. These often rely on multilevel frameworks like Norris et al^24^ that address the discursive, legal, and cultural dimensions of stigma. Six studies^41,45,48,52,55,56^ have drawn on general stigma frameworks, such as Link and Phelan,^59^ or Shellenberg,^60^ to conceptualise broader social processes.

##### Structural and sociocultural approaches

Two more recent studies^48,57^ have referenced critical and structural approaches, such as Letourneau, Millar and Ratcliffe.^26,61,62^ In doing so, they conceptualise abortion stigma as a sociocultural process shaped by power relations and structural inequities,^26,62^ and highlight the legal, cultural, and religious drivers operating at the institutional level.^62^

#### Types and dimensionalities of stigma

##### Typological approaches

Three studies^38,56,57^ used the established typology of internalised, felt, enacted stigma according to Cockrill and Nack.^58^

##### Single-entity views

Two studies used less nuanced accounts. Smith et al^52^ offered a community-level definition of abortion stigma as “social disapproval,” which was adopted by Rice et al,^39^ thereby failing to clearly specify the drivers and mechanisms of stigma, resulting in a partial alignment between definition and analytic scope.

##### Multidimensional/structural views

Definitions drawing on multidimensional or multilevel frameworks, such as those by Letourneau et al,^61^ Norris et al,^24^ Millar,^26^ and Ratcliffe^62^ proved particularly useful for analysing stigma across individual, interpersonal, and structural levels, as seen in studies like Giovannelli et al^56^ and Bloomer et al^57^

##### No definition

Four studies^47,49,50,54^ lacked an abortion-specific definition limiting their conceptual clarity and weakening interpretive power.

#### Stigma drivers and power dynamics

##### Gender norms and ideal of womanhood

Eleven studies^18,36–38,40,42,48,53,55–57^ referencing Kumar et al's definition frame stigma as arising from deviation from the cultural expectations of femininity and reproduction, positioning abortion seekers as socially deviant. Here, power is implicit, operating through the regulation of womanhood and sexuality.

##### Legal, religious, and cultural regimes

Three studies^37,40,45^ applied Norris et al's^24^ definition and one study^56^ referred to Letourneu et al,^61^ highlighting law, fetal personification, religion, and cultural authority as institutional drivers of stigma. Power is exercised through these regimes, legitimising stigma across social and structural levels.

##### Discursive and normative processes

Two studies^48,57^ referencing Millar^26^ and Ratcliffe et al^62^ define stigma as a sociocultural process tied to categories of difference, with discourses constructing abortion as deviant. Stigma functions as a classificatory practice that maintains inequality and reproduces social power.

##### Typological manifestations without drivers

Three studies^38,56,57^ used the definition proposed by Cockrill et al,^58^ who differentiate between internalised, felt, and enacted manifestations of abortion-related stigma at an individual level. This approach captures personal experiences but leaves drivers and power unexplored.

### RQ2: Abortion stigma among the public (quantitative studies)

We included seven quantitative studies in the analysis.^36–42^ Six studies were conducted in the USA and one in Germany. Five studies investigated stigma among the general public,^36–38,41,42^ while two focused exclusively on women.^39,40^ Table 1 provides an overview of the study origins, sample sizes, measurement instruments, and associations between stigma levels and the relevant factors.

**Table 1:** Overview quantitative studies; study origins, sample sizes, measurement instruments, and associations between stigma levels and the relevant factors

Study	**Abortion-stigma** **measurement tool**	Prevalence (%)/ level of stigma (Mean, SD)	Explored associations with correlations
**Bommaraju (2016) [22]^2^** Country: USA Sample: n=306	Abortion Stigma Perception (ASP) • α=0.86 • 8 items • Scoring: 4-point Likert Scale, higher scores indicate stronger stigma; score range: 8-32	ASP M=21.0 (SD=4.4)	- Non-Hispanic white women held higher abortion stigma perceptions scores than non-Hispanic Black (b=2.58; 95% CI: 1.33–3.84])
**Rice (2017) [20]^1^** Country: USA Sample: n=642	Abortion Norms and Stigma Scale • α=0.94 • 21 items • Scoring: 5-point Likert scale, higher scores indicate stronger stigma; score range: 1-5 • 4 Subscales: - Conditional Acceptability Scale (4 items, α=0.94) - Anticipated Reactions Scale (7 items, α=0.88) - Misperception Scale (2 items, α=0.81) - Attitudes Scale (8 items, α=0.90)	ANSS Total score M=3.63 (SD=0.86) Conditional acceptability M=3.49 (SD=1.20) Anticipated reactions M=4.02 (SD=0.94) Misperception M=4.06 (SD=0.99) AttitudesM=3.26 (SD=1.09)	- Religiosity was associated with higher abortion stigma (b=0.46; SE=0.07, p<0.001) - Women who identify as “other” race (compared to white, showed less abortion stigma (b=-0.34; SE=0.10, p<0.01
**Patev** (**2019)[23] ^2^** Country: USA Sample: n=303	Stigmatizing Attitudes, Beliefs, and Actions Scale (SABAS) • α=0.93 • 18 items • Scoring: 5-point Likert Scale • 3 subscales: - Negative stereotyping - Exclusion and discrimination - Contagion Abortion legality attitudes • α=0.87 • Scoring: 5-point-Likert scale; lower scores indicate more negative attitudes toward abortion legality • 3 items: - Abortion is wrong, because everyone, even unborn babies have the right to life - Abortion should be illegal - It’s a woman’s constitutional right to choose whether or not to have an abortion	SABAS women: M=32.82, SD=13.25 SABAS men: M=37.52, SD=13.77	Gender - Higher stigma scores in men compared to women t(303)=3.03, p=.003 Men - men who report less SABAS, those who identify as religious had more negative attitudes toward abortion legality than those who identiﬁed as not religious [B (SE)=3.18(0.76), p<.001]. - men who reported more SABAS, religiosity did not inﬂuence their attitudes toward abortion legality [B(SE)=0.46(0.64), p=.47] Women - women who report less SABAS, those who identify as religious had more negative attitudes toward abortion legality than those who identiﬁed as not religious [B (SE)=1.40(0.53), p=.009]. - women who identify as religious had more negative attitudes toward abortion legality than those who identiﬁed as not religious [B(SE)=2.11(0.74), p=.005] Religiosity - Religiosity associated with more negative attitudes toward abortion legality [B(SE)=1.84(0.33), p<.001] - SABAS were negatively associated with abortion legality attitudes [B(SE)=−0.11(0.01), p<.001] Interaction - signiﬁcant interaction between abortion stigmatizing attitudes, religiosity, and gender, [B(SE)=0.11(0.05), p=.01]
Hanschmidt (2020) [19] ^1^ Country: GER Sample: n=14,459	Support for abortion access in case of … - … fetal health risk - … no more children - … woman’s health risk - … socio-economic restrictions - … rape - … single woman	Eastern GER Unrestricted support 1992: 75% 2012: 59.9% Partial support 1992: 19.6% 2012: 40.2% No support 1992: 0.9% 2012: 3.2% Western GER Unrestricted support 1992: 47% 2012: 31% Partial support 1992: 48.9% 2012: 58.9% No support 1992: 4.1% 2012: 10.1%	Time trends - Over the study period (1992–2012), public support for abortion restrictions increased in both Eastern and Western Germany Sociodemographic factors - Religiosity was associated with restrictive abortion attitudes - Identifying as political left-wing, being employed, being female, and having higher education were less likely to have restrictive abortion attitudes Barriers to abortion care - Regions with higher state-level barriers to abortion (few abortion facilities per woman and a high proportion of women seeking out-of-state abortions) were associated with more restrictive abortion attitudes
**Cutler (2021) [18]^1^** Country: USA Sample: n=886	Three dimensions of abortion stigmaJudgement: • Community Abortion Attitudes Scale (CAAS); α=0.91 • 7 items • Scoring: 5-point Likert scale; scores ≥ 3 indicating high stigma Context: • Abortion subscale of Reproductive Experiences and Events Scale (REES); α=0.87 • 5 items • Scoring: 100-point scale, higher scores indicating more positive feelings Secrecy: • Silence Subscale of the Community Level Abortion Stigma Scale (CLASS); α=0.78 • 4 items • Scoring: 5-point Likert scale, higher scores indicating more stigma Support of abortion policies General support of abortion-related policies • α=0.913 • 6 items) • Scoring: scale 1-4 Conditional support of abortion-related policies • α=0.80 • 3 items • Scoring: scale 1-3	CAAS M=2.17 (SD=0.93) REES M=46.95 (SD=27.41) CLASS M=3.08 (SD=0.84) General Support for Abortion Policies Median:2.33 (IQR:1.63–2.95) 67% “less favorable” attitudes Conditional Support for Abortion Policies Median= 2.73 (IQR: 2.32–2.86) 40% “less favorable” attitudes	CAAS Religion (vs. none) - Catholic: OR = 2.97 (95% CI: 1.49–5.91) - Evangelical or Protestant: OR = 4.78 (95% CI: 2.53–9.00) Party Affiliation (vs. Democrat) - Republican: OR = 11.56 (95% CI: 6.21–21.52) - Independent: OR = 5.80 (95% CI: 3.17–10.60) REES Religion (vs. none) - Catholic: OR = 3.08 (95% CI: 1.43–6.65) - Evangelical or Protestant: OR = 4.34 (95% CI: 2.15–8.75) Party Affiliation (vs. Democrat) - Republican: OR = 6.33 (95% CI: 3.41–11.74) - Independent: OR = 2.75 (95% CI: 1.51–4.99) **CLASS** Religion (vs. none) - Catholic: OR = 1.66 (95% CI: 1.02–2.68)Race/ethnicity (vs. Non-Hispanic White) - Black race: OR = 1.91 (95% CI: 1.09–3.32) - Other race: OR = 2.19 (95% CI: 1.05–4.54) Gender (vs. male) - Female respondents: OR = 0.66 (95% CI: 0.47–0.91) - Reproductive History (vs. yes) - No personal or partner pregnancy history: OR = 0.63 (95% CI: 0.42–0.95) General support Religion (vs. none) - Catholic: OR = 5.82 (95% CI: 3.29–10.28) - Evangelical or Protestant: OR = 5.82 (95% CI: 3.48–9.72) - Other religion: OR = 3.38 (95% CI: 1.84–6.18) Party affiliation (vs. Democrat) - Republican: OR = 6.52 (95% CI: 3.62–11.70) - Independent: OR = 2.51 (95% CI: 1.65–3.83) Income (vs. > $125,000) - Income < $40,000: OR = 3.01 (95% CI: 1.56–5.80) - Income $40,000 - $75,000: OR = 2.51 (95% CI: 1.65–3.83) Reproductive History (vs. yes) - Lack of proximity to someone who had an abortion: OR = 2.57 (95% CI: 1.47–4.47) - No biological children: OR = 0.50 (95% CI: 0.32–0.78) (less likely to have unfavorable views) Conditional support Religion (vs. none) - Catholic: OR = 1.78 (95% CI: 1.06–2.99) - Evangelical or Protestant: OR = 2.98 (95% CI: 1.82–4.87) - Other religion: OR = 2.03 (95% CI: 1.13–3.66) Party Affiliation (vs. Democrat) - Republican: OR = 4.02 (95% CI: 2.53–6.39) - Independent: OR = 2.29 (95% CI: 1.51–3.47) Income (vs. > $125,000) - Income < $40,000: OR = 2.25 (95% CI: 1.32–3.83) - Income $40,000 - $75,000: OR = 2.65 (95% CI: 1.61–4.37)
**Cutler (2022) [24]^3^** Country: USA Sample: n=735	Three dimensions of abortion stigmaJudgement: - Community Abortion Attitudes Scale (CAAS)Context: - Abortion subscale of the Reproductive Experiences and Events Scale (REES)Secrecy: - Silence Subscale of the Community Level Abortion Stigma Scale (CLASS)	Not reported	Intervention group vs. control group - Intervention group: no decreased score (CAAS & CLASS) immediately [OR, 0.80 [95% CI, 0.59–1.09] and [OR, 1.28 [95% CI, 0.93–1.75], respectively) or at the 3-month follow-up [OR, 0.86 [95% CI, 0.62–1.19] and [OR, 0.98 [95% CI, 0.70–1.37] compared with controls - Intervention group: decreased stigma (REES) immediately after the intervention [OR, 1.74; 95% CI, 1.23–2.46]; but not at the 3-month follow-up [OR, 0.98; 95% CI, 0.70–1.37]
**Stowers (2023) [21]^1^** Country: USA Sample: n=911	Community-Level Abortion Stigma Scale (CLASS) • α=0.9 • 23 items • Scoring: 5-point Likert scale; higher scores indicate more stigma • 4 subdomains: - Autonomy (4 items) - Stereotyping (11 items) - Religion (4 items) - Secrecy (4 items)	CLASS Total Score M=2.49 (SD=0.9) Subscales: Autonomy: (M=2.63, SD=0.9) Stereotyping M=2.29 (SD=1.10) Religion: M=2.41 (SD=1.28) Secrecy: M=2.97 (SD=0.96)	Belief in a just world - Stronger belief in a just world is associated with higher abortion stigma [(β=0.70 / Standardized β=0.30 (95% CI: 0.58–0.82)] Gender - Men report significantly higher abortion stigma compared to women [β=4.14 / Standardized β=0.10 (95% CI: 2.22–6.05)] Race - Asian respondents report significantly lower abortion stigma compared to other racial groups [β=-7.21 /Standardized β=-0.10 (95% CI: -10.68–(-3.75))] Religion - Stronger religious beliefs are strongly associated with increased abortion stigma [ β=0.26 / Standardized β=0.46 (95% CI: 0.23–0.29)] Reproductive history - Having pregnancy experience (self or partner) is linked to higher abortion stigma [β=3.07 / Standardized β=0.08 (95% CI: 1.09–5.04)] Education - Higher education (beyond college degree) is positively associated with abortion stigma [β=2.75 / Standardized β=0.06 (95% CI: 0.59–4.09)] Insignificant associations - No association between abortion stigma and age, having previous abortion, black race, white race

*Note* 1 ROBINS-Score (Risk of bias): 1 = low risk 2 = some concerns 3 = high risk.

#### Stigma dimensions

The included studies applied a range of instruments to measure different dimensions of abortion stigma among the public. For analytical clarity, we grouped the scales and subscales into six overarching stigma dimensions: (a) perceived stigma, (b) enacted stigma, (c) attitudes/stereotypes, (d) acceptability, (e) anticipated stigma, and (f) misperceptions. These categories capture the different ways in which stigma manifests, ranging from societal judgment to discriminatory attitudes and misinformation. While some instruments combined multiple aspects of stigma within the same (sub)scale, we categorised them based on the predominant focus of the items. This approach allows for a clearer comparison across studies while acknowledging that some measures cross conceptual boundaries (cf. Supplementary Table 5)

- *Perceived stigma* refers to individuals’ awareness of societal judgments surrounding abortion, even in the absence of direct confrontation. This dimension was captured using the Abortion Stigma Perception (ASP) scale. This eight-item scale measures how strongly individuals perceive societal disapproval and judgment toward people who have had an abortion. Only one study applied the ASP and yielded a moderate mean score among postpartum women in the USA (*M* = 21.0, SD = 4.4; possible score range 8–32),^40^ suggesting a widely shared perception that abortion is socially disapproved.
- *Enacted stigma* refers to actual discriminatory behaviour or the endorsement of exclusionary practices. This dimension was measured exclusively through the Exclusion and Discrimination subscale of the Stigmatising Attitudes, Beliefs, and Actions Scale (SABAS).^41^ This subscale measures the extent to which respondents endorse exclusionary behaviour or punitive actions toward women who have had an abortion. Although the SABAS includes an enacted stigma subscale, the respective study^41^ reported only overall scores, making a subscale-level analysis impossible.
- *Attitudes and stereotypes*, combining general evaluations and moral or character-based judgments. Cutler et al^36^ used the Community Abortion Attitudes Scale (CAAS) that assesses general attitudes about abortion and the moral character of those who have abortions. Given that scores of 3 or higher reflect high stigma on the CAAS, the observed mean of 2.17 (SD  =  0.93) indicated relatively low abortion stigma among participants.^36^ Stowers et al^38^ used the Community-Level Abortion Stigma Scale (CLASS), which includes a subscale measuring stereotypical and stigmatising views about women who have abortions, such as being seen as irresponsible or undeserving of family life. They reported a mean score of 2.29 (SD  =  1.10) on a scale ranging from 1 to 5.^38^ They also applied the Religion subscale of the CLASS, which captures morally and religiously motivated judgments of abortion. As these items reflect socially shared value-based attitudes, rather than perceived or anticipated stigma, we categorised this subscale as attitudes/stereotypes. Results indicated a moderate level of religiously framed stigma (*M*  =  2.42, SD  =  1.28). Rice et al^39^ applied the Attitudes subscale of the Abortion Norms and Stigma Scale. This subscale captures moral evaluations and character-based judgments toward abortion and those who have abortions, such as seeing them as selfish, immature, or cold. The results indicated a moderate level of stigmatising attitudes (*M*  =  3.26, SD  =  1.09 on a 5-point scale), suggesting that such stereotypes remain prevalent in public discourse.
- *Acceptability*, understood as the moral, situational, or legal acceptance of abortion, was assessed using multiple instruments. For example, Rice et al^39^ used the Conditional Acceptability subscale of the Abortion Norm and Stigma Scale to capture whether abortion is considered acceptable under specific life circumstances, such as lack of support or difficult living conditions. They reported high scores on this subscale (*M*  =  3.49, SD  =  1.20, score range 1–5), indicating less conditional acceptability of abortions. Stowers et al^38^ measured participants’ view on autonomy on their decision to terminate a pregnancy using the Autonomy subscale of the CLASS, which measures the degree to which abortion is viewed as a legitimate and responsible personal choice. They found moderate levels of stigma (*M* = 2.63, SD = 0.99, score range 1–5), with higher scores indicating a higher degree of stigmatising attitudes. Three studies assessed participants’ views on abortion policy support; however, only two of them reported the corresponding mean scores or prevalence data.^36,37,41^ According to Cutler et al,^36^ 67% of participants showed low support for general abortion policies and 40% expressed similarly unfavourable attitudes toward conditional abortion access. Hanschmidt et al^37^ analysed trends in Germany, demonstrating a decline in unrestricted support for abortion over time. In Eastern Germany, support decreased from 75% in 1992 to 59.9% in 2012, while in Western Germany, it dropped from 47% to 31%.
- *Anticipated stigma*, defined as the expectation of rejection or disapproval if one’s abortion became known, was measured using two subscales. The Anticipated Reactions Scale captures expected emotional and social responses from close others, while the Secrecy subscale of the CLASS reflects the belief that abortion should be kept hidden from others. Rice et al^39^ reported that anticipated reactions received high ratings (*M*  =  4.02, SD  =  0.94, score range: 1–5), indicating that many participants feared negative responses from their social environment. Cutler et al^36^ also found relatively high scores on the Secrecy subscale (*M*  =  3.08, SD  =  0.84), suggesting that abortion remains a social taboo and is often seen as something that should be kept hidden.
- f. *Misperceptions*, including false beliefs about health or social consequences, were assessed by Rice et al^39^ using the two-item Misperception Subscale of the Abortion Norms and Stigma Scale. It measures belief in medically inaccurate claims, such as the idea that abortion harms future fertility or health. The results revealed a high level of misperception (*M*  =  4.06, SD  =  0.99; scale range: 1–5), suggesting that misinformation continues to play a key role in reinforcing abortion stigma. Similarly, the Fear of Contagion subscale of the SABAS captures highly stigmatising and inaccurate beliefs, such as that abortion could cause illness or social contamination. This subscale was included in Patev et al^27^; however, no subscale-level data were reported, and only the total SABAS score was available.

### RQ3: Associated factors with abortion stigma (quantitative studies)

In addition to reporting stigma levels across different dimensions, six studies have also examined which factors are associated with higher or lower levels of abortion stigma. Sociodemographic and ideological factors have been frequently examined as predictors of stigma. The following associations emerged across the studies.

#### Religiosity

Religiosity has consistently been identified as one of the strongest predictors of higher abortion stigma across multiple studies and instruments. Rice et al^39^ found that religiosity was significantly associated with higher stigma scores on the Abortion Norms and Stigma Scale (*b*  =  0.46; SE  =  0.07; *p* < .001). Patev et al^41^ reported that religious affiliation correlated with more negative attitudes toward abortion legality, particularly among participants with lower overall SABAS scores. Stowers et al^38^ reported a significant association between stronger religious beliefs and increased abortion stigma using the CLASS. Cutler et al^36^ linked religious identity to increased stigma across three CLASS subscales: those identifying as Catholic or Evangelical/Protestant were more likely to hold judgmental attitudes (CAAS), endorse secrecy (CLASS), report negative emotional responses (REES) and show less support for abortion-related policies. Similarly, Hanschmidt et al^37^ found that religiosity was associated with more restrictive attitudes towards abortion in a large German sample. Taken together, these findings underscore that religious belief systems, particularly those grounded in conservative frameworks, continue to shape stigmatising views on abortions.

#### Political orientation and policies

Political affiliation was another strong and consistent predictor of abortion stigma. Cutler et al^36^ found that Republican and Independent participants were significantly more likely than Democrats to hold stigmatising attitudes in the USA. This included general and conditional support for abortion policies, judgmental attitudes (CAAS: Republican OR  =  11.56) and negative affective responses (REES: Republican OR  =  6.33). In Germany, Hanschmidt et al^37^ found a similar, although less pronounced, pattern: individuals who identified with left-wing political positions were less likely to express restrictive attitudes toward abortion. These findings suggest that political ideology, particularly conservative or right-leaning positions, is closely linked to higher levels of abortion stigma, possibly reflecting broader ideological views of gender, family, and reproductive rights.

One study linked public attitudes toward abortion stigma with policy and service availability: Hanschmidt et al^37^ found that opposition to abortion was strongest in regions with fewer facilities and greater barriers to care, illustrating how structural restrictions on access both reinforce and legitimise stigmatising public attitudes.

#### Gender

Two studies reported higher stigma scores among men than women.^38,41^ Patev et al^38,41^ found significantly higher overall stigma among men using the total SABAS score (t(303) = 3.03, *p* = .003), while Stowers et al^38^ observed similar results using the CLASS scale (*β* = 4.14; standardised *β* = 0.10; 95% CI: 2.22–6.05). Interestingly, Cutler et al^36^ found that men were more likely than women to agree that abortion should be kept secret, as indicated by higher scores on the Secrecy Subscale of the CLASS (OR = 0.66; 95% CI: 0.47–0.91).

#### Race and ethnicity

Findings on race and ethnicity differed depending on the stigma dimension measured: while Bommaraju et al^26^ found that non-Hispanic White women reported more perceived societal stigma than non-Hispanic Black women (ASP (*b* = 2.58; 95% CI: 1.33–3.84)), findings on anticipated stigma, as measured by the Secrecy subscale of the CLASS, indicated that Black participants were more likely than non-Hispanic White respondents to feel the need to conceal an abortion (OR = 1.91, 95% CI: 1.09–3.32).^36^ Stowers et al^38^ analysed the total CLASS Scale comprising four subscales that reflect different forms of abortion stigma: autonomy, stereotyping and discrimination, religion, and secrecy. They reported lower stigma scores among Asian participants than among other racial groups (*β* = −0.10; 95% CI: −10.68–(−3.75)); however, they found no differences between Black and White participants.^38^ Using the Abortion Norms and Stigma Scale, which captures multiple dimensions of stigma, Rice et al^39^ found that non-Hispanic White women reported higher levels of abortion stigma than women of “other” racial backgrounds (excluding Black participants).

#### Socioeconomic status and education

Socioeconomic factors further influence stigma, as lower-income individuals tend to report more restrictive abortion attitudes,^36^ whereas higher education levels are typically linked to lower stigma.^37^ However, some findings suggest that advanced education does not always correlate with greater acceptance of abortion.^38^

#### Reproductive history and proximity to abortion

Participants who had close contact with someone who had an abortion or who had no biological children tended to report lower levels of abortion stigma.^36^ In contrast, Stowers et al^38^ indicated that individuals who had experienced pregnancy themselves or through a partner were more likely to hold stigmatising views.

### RQ4: Lived experiences concerning abortion stigma (qualitative studies)

The qualitative studies included in this review were conducted in a range of countries. Five studies were conducted in the USA,^47–50,63^ followed by two studies each from Canada^54,55^ and Australia.^18,53^ Additionally, three studies were conducted in European countries, specifically the United Kingdom,^52^ Italy,^56^ and the Republic of Ireland and Northern Ireland.^57^ Qualitative studies show a high diversity in possible study populations and designs. The synthesis of qualitative findings reveals that abortion stigma in HICs manifests across multiple social levels and takes diverse forms.

Our sample includes studies focusing on the general public^48,49,52,57^ as well as on specific groups within the public, such as religious leaders,^50,51^ anti-abortion activists,^54,55^ pro-choice activists,^56^ and journalists.^47^ In addition, two studies analysed (social) media to illustrate abortion stigma in the public eye.^18,53^ We found components of enacted stigma^47–50,52–57^ and perceived stigma^47,56^ in all articles. In addition, we identified thoughts on mitigating factors^18,48–50,54–56^ of abortion stigma among the general public.

#### Enacted stigma

The review identified studies in which social groups were targets of enacted stigma, as well as studies showing that members of the general public actively enacted stigma toward individuals associated with abortion. Across the included studies, participants from various backgrounds reported experiencing enacted stigma in different forms, including verbal harassment, discrimination, and social exclusion. For example, pro-choice activists reported direct verbal attacks during public demonstrations^56^:

“*I remember they shouted at me insults such as ‘whore’ and so on. […] Another time, I was with other activists and we had banners in our hands. I remember that the people who manage the security service hurled at us grave offenses.*” (p.7)

“*Once, in front of an Italian hospital, there was a prayer group against abortion. These people pushed us. It was a bit unexpected; a minute before they were praying and immediately after they started shoving us.*” (p.8)

Journalists covering abortion also experienced harassment, including threats and public exposure of private information:

“*Antis [anti-abortion advocates] tweeted out my home address. So that was an issue for me as a writer and it did have a chilling effect … It made me really terrified.*”^47^ (p.398)

Similarly, abortion providers were described as ignorant and morally corrupt, perceived as participating in harm rather than care:

“*They probably feel like they’re helping a lot of women. […] I can’t judge them for their ignorance. I’m glad they want to help women. I wish they knew more about what they were actually doing.*” ^55^ (p.454)

Among religious leaders, enacted abortion stigma often manifested through moral discourses that frame abortion as sinful and deserving of divine punishment. For instance, some participants described abortion as a violation of religious tenets, with one pastor asserting that women who have abortions

“*[…] ‘just get paid back by the Lord’.*”^50^ (p.42)

While several studies in this review focus on specific groups within the public, Baker et al^48^ was the only study that identified explicit forms of enacted abortion stigma within a general public sample. The findings demonstrate that members of the general public not only hold stigmatising views but also express them through direct calls for punitive measures against women who seek abortions and those involved in providing abortion care.

“*Sterilization, as an extreme … if they made a bad decision and they just don’t want the kid, I think that they need to be sterilized*.” (p.41)

“*A lot of things come to mind, to say there should be fines, but also a punishment, jail, because it is a murder. For life, for murder, that is why these people should be put in jail*.” (p.40)

“*I like what Alabama or Georgia, I think, set up … a system where doctors who do it would be punished, and I think that’s the right way to do it … make it the same as the rest of your penal system for homicide*.” (p.40)

Although these examples illustrate direct forms of enacted stigma, perceived and anticipated stigma are also present in the analysed studies, influencing how individuals experience and manage abortion stigma in more subtle yet persistent ways.

#### Perceived stigma

Perceived stigma refers to the awareness and recognition of negative attitudes and beliefs about abortion, either attributed to the public or held personally. Many participants described how they perceive dominant public attitudes toward abortion, often marked by moral judgment and strong condemnation. For instance, pro-choice activists reflected on how they believe they are perceived by society, emphasising the hostile ways in which both women who have abortions and abortion rights advocates are viewed:

“*[The pro-choice activists] are seen as Satan. […] There is just the feeling that people perceive them as a bugbear, a devil. Women who choose to have an abortion are seen as sinners and activists as witches, the antichrist. There is this medieval perception of women*.”^56^ (p.10)

But young women from the general public also reflected on how society views abortion, often reporting a hostile and stigmatising environment. One participant described her community’s perception of abortion as morally unacceptable and associated with “killing a child”:

“*Well like I’m from deep in the south … They kind of view it as a bad thing like you’re killing your child. That’s why most people in my community do keep their children because they really don’t want to kill a baby*.”^52^ (p.11)

This reflects the strong stigmatisation of abortion within the community, which leads many women to conceal their abortion experiences out of fear of judgment. As another participant noted:

“*There’s probably more women that have had [an abortion], but it’s something they might be ashamed of and don’t tell anyone*.”^52^ (p.10)

These statements illustrate how perceived public stigma shapes individual behaviour, contributing to the silencing and invisibility of abortion experiences. Negative public perceptions of abortion were also reinforced by anti-choice participants, who emphasised public condemnation of abortion and those involved in it.

“*If you were to do something like that [get an abortion and] if your family finds out and then they think differently of you, I mean, you deserved that. You put yourself in that situation*.”^48^ (p.42)

In addition to these public perceptions, participants’ statements also highlight individual perceptions of abortion, shaped by internalised moral, religious, and cultural beliefs.

Individuals holding anti-abortion attitudes described women who seek abortions as morally flawed and in need of correction:

“*Participants characterized women seeking abortion as having made ‘bad decisions’ and needing to ‘get fixed*’.” ^48^ (p.41)

Negative individual perceptions of abortion are reinforced by media discourses that reproduce a deviant image of womanhood:

“*… good-hearted women whose inner lives have been wounded by abortion – having created a place of death in their body*.”^53^ (p.760–761)

Moreover, in the context of workplace discussions, abortion was also framed as a selfish lifestyle choice, detached from legitimate reproductive health needs. For instance, participants expressed concern that relaxing abortion laws would normalise abortion as a routine method of contraception, driven not by rights but by individual convenience:

“*Any relaxing of legislative restrictions would begin ‘normalizing abortion as a form of contraception’, the demand for which is driven not by reproductive rights but ‘to protect lifestyles, imagine sacrificing a baby's life to protect a lifestyle*’.”^57^ (p.12)

Finally, a young woman expressed her individual negative perceptions of abortion, describing abortion as inherently selfish and unacceptable:

“*I think it’s horrible. I just wish they never created abortion clinics – the word abortion … I wish it never existed. I think it’s ridiculous. I think it’s all selfish*.”^52^ (p.11)

#### Anticipated stigma

The findings also highlight the role of anticipated abortion stigma, which refers to the fear of experiencing negative reactions or judgment if one’s abortion becomes known. This form of stigma leads individuals to avoid disclosure and silence their experiences, especially in sensitive social contexts. For instance, women reported concealing their abortion in the workplace due to fear of stigmatisation, as illustrated by one participant:

“*I worked with a colleague who made the decision to have an abortion … they did not disclose this information to our employer as they were so worried about the stigma. Instead my colleague took sick leave to travel to England for their procedure*.”^57^ (p.9)

Similarly, pro-choice activists emphasised how anticipated stigma shapes their behaviour in professional environments, such as academia, where they refrain from revealing their engagement in abortion-related activism:

“*I felt that my activism would be seen as a negative thing … I’m not sure, but this was my gut feeling and, therefore, for this reason, I did not speak about it*.”^56^ (p.13)

These findings demonstrate how anticipated stigma reinforces the broader social silencing of abortion and contributes to its invisibility in everyday life.

#### Mitigating factors

Although abortion stigma is pervasive across individual, interpersonal, and societal levels, several mitigating factors emerged from the included qualitative studies. These factors illustrate how individuals and communities seek to counteract stigma and promote supportive environments for people who experience or are associated with abortion.

One central strategy to mitigate stigma is the active creation and dissemination of counter-narratives, particularly by pro-choice activists. These narratives aim to normalise abortion and emphasise women’s autonomy and decision-making power. For example, participants in Giovannelli et al^56^ described how speaking openly about abortion and their activism was not only an act of resistance but also a way to reclaim their identity and counter public stigma:

“*I speak openly about it [activism] even if I know there is a prejudice. My ideas have always been quite clear […]. I have never done any kind of censorship and I prefer to talk because I think it [activism] is a part of me, an added value of my person*.”^56^ (p.12)

Such open discourse and visibility were seen as essential in challenging the silence that typically surrounds abortion and in empowering others to share their experiences. Another important mitigating factor was found in supportive professional and activist communities, such as trade unions or pro-choice groups, where individuals could share experiences without fear of judgment. Bloomer et al^57^ highlight how union-led discussions about abortion helped to reduce stigma in professional contexts and enabled participants to see abortion as a legitimate workplace issue:

“*Following the focus group activity, some participants modified their perspectives on the issue, with open discussions viewed as a key component in ‘normalizing and de-stigmatizing abortion’*.”^57^ (p.11)

In addition to activist spaces, reproductive health providers play a crucial role in mitigating stigma by framing abortion as routine medical care. Clinic websites analysed by Baird and Millar^18^ emphasised patient-centred care and autonomy, rejecting stigma-laden narratives by positioning abortion as a normal part of healthcare:

“*Our philosophy is to remove that stigma, by making the procedure as acceptable and respected as any other gynaecological operation*.”^18^ (p.7)

Such clinical framing is vital to countering the societal notion of abortion as a deviant or morally wrong act. Finally, supportive religious perspectives – though less prevalent – also emerged as potential mitigating factors. Some religious leaders emphasised compassionate, non-judgmental pastoral care, focusing on understanding rather than condemnation. As one participant in Dozier et al^50^ stated:

“*My personal views on abortion? Hmm … That’s a decision that the individual and God should make*.”^50^ (p.1524)

Another pastor reflected recognising both the woman’s right and the value of potential life:

“*I have no right to say to someone who’s carrying a child that you can or cannot do this […] but at the same time, there is also the potential for a life being carried inside of that body*.”^50^ (p.8)

This approach contrasts with dominant religious discourses that often perpetuate stigma and illustrates the possibility of creating more inclusive and understanding faith-based responses.

Overall, these findings underscore that collective spaces, supportive discourse, professional framing, and compassionate religious counselling can play a critical role in challenging and mitigating abortion stigma. Such efforts highlight the importance of contextual, relational, and structural interventions in reducing the harmful effects of stigma on individuals and communities.

### Integration of quantitative and qualitative findings

Synthesising the quantitative and qualitative findings reveals a set of converging patterns regarding the nature, expression, and impact of abortion stigma among the public in HICs. First, both bodies of literature underscore the pervasiveness and persistence of abortion stigma, despite varying cultural and national contexts. Quantitative data demonstrate moderate yet enduring levels of stigma in the public^36,38^ with interventions showing only short-term effectiveness.^42^ Correspondingly, qualitative accounts reveal how abortion stigma is socially embedded and sustained through mechanisms of silence, moral judgment, and fear of disclosure.^48,56,57^

Second, both quantitative and qualitative findings emphasise that abortion stigma operates across multiple social levels. Quantitative studies capture these layers through validated instruments that assess dimensions such as secrecy, judgmental attitudes, and discrimination.^38,41^ In parallel, qualitative studies provide rich accounts of how stigma is enacted by others (e.g. harassment or verbal attacks), perceived through societal judgment, and anticipated in everyday interactions, such as at work or within families.^48,56^ This alignment demonstrates that stigma is not limited to individual beliefs but is deeply rooted in social relationships and institutional structures.

Third, quantitative studies identify demographic and ideological factors – such as religiosity,^36,38,41^ political conservatism,^36,37^ sex/gender,^36,41^ and socioeconomic status^36–38^ – as key predictors of abortion stigma.^37,41^ While qualitative studies do not systematically examine these variables, they provide contextual support for some of these patterns. For instance, accounts from religious leaders^50,51^ and individuals in conservative communities^52^ reflect how ideological worldviews shape stigma expressions. However, detailed analyses of gendered or class-based differences are largely absent from the qualitative data, highlighting a potential area for future research.

While the methods yielded distinct insights, these categories highlighted the shared dimensions of how stigma is enacted and perceived, emphasising the interplay of societal norms, individual experiences, and structural barriers.

## Discussion

This review is part of a larger project that explores abortion stigma from three perspectives: those who have abortions, those who provide abortions, and the public. With this review, we aimed to update the evidence on the prevalence and forms of abortion stigma among the public. In line with the findings of Hanschmidt et al,^7^ the updated review also revealed that only a limited number of studies have addressed abortion stigma among the public. Most studies (11 in total) originated from the USA. Only eight studies were conducted outside the USA – four in Europe, two in Australia, and two in Canada. This uneven distribution makes cross-country comparisons challenging and underscores the need for further research in diverse settings. Further, the studies included in our review encompassed heterogeneous samples, ranging from the general public to more specific groups such as pro-choice and anti-abortion activists, journalists, religious leaders, and even posts on social media platforms. This diversity makes it difficult to draw comprehensive or generalised conclusions about the findings. However, one key observation that emerges across the different studies is that abortion stigma remains persistently widespread and operates at multiple levels.

### Updated evidence

Hanschmidt et al^7^ included three quantitative and two qualitative studies in their review. Their findings indicate that public attitudes toward women with a history of abortion are often characterised by stigmatisation, particularly in terms of social rejection, negative stereotyping, and moral condemnation. The studies they reviewed suggested moderately strong to pronounced levels of stigma, with religious and moral beliefs playing a central role. Our updated analysis confirms these fundamental patterns while offering a more nuanced and comprehensive perspective on abortion stigma. By incorporating a broader range of methodologies – including social media analyses and longitudinal studies – this review captures the complex and evolving nature of public attitudes and the multiple levels at which abortion stigma operates. Our research indicates that in HICs, the stigma surrounding abortion remains at a moderate level but continues to persist. Factors such as gender, political affiliation, and socioeconomic status play a significant role in shaping this enduring stigma. While Hanschmidt et al^7^ primarily documented explicit rejection and negative stereotyping, our analysis also emphasised aspects such as secrecy and conditional acceptance of abortion. This suggests that stigmatisation is not only expressed through overt prejudice but also through subtle mechanisms such as social silence or symbolic language.

### Abortion stigma as a structural and human rights issue

Our findings show that abortion stigma is not only enacted at the interpersonal level, but is also deeply embedded in legal, institutional, and discursive structures. One included German study^37^ indicated that regions with fewer abortion facilities and greater barriers to care tend to show stronger opposition to abortion, suggesting that limited access may reinforce restrictive attitudes rather than simply reflect them. Similarly, religiosity and political conservatism emerged as some of the strongest predictors of higher abortion stigma, underlining how ideology and power relations shape public opinion.^36–39,41^ One qualitative study further illustrated how media discourse,^53^ religious narratives,^50^ and anti-abortion activists^54,55^ sustain a climate of condemnation in which abortion is framed as morally deviant and those associated with it are devalued. These examples highlight that abortion stigma operates through a self-perpetuating cycle of structural stigma, in which legal restrictions and public attitudes mutually reinforce one another.

From a human rights perspective, these findings carry important implications. In international human rights frameworks, abortion is recognised as part of the fundamental right to health and bodily autonomy.^3,11,12^ However, this recognition is not universal, and even in settings where abortion is legally permitted, persistent public stigma and structural barriers continue to limit the realisation of this right, particularly for individuals living in restrictive or conservative environments.

Enacted stigma in the form of harassment, discrimination, and threats violates the right to dignity and security.^64^ Perceived and anticipated stigma, which may lead individuals to silence their experiences and avoid disclosure, can also restrict their opportunities for free expression and participation in public life. Structural stigma in the form of restrictive laws or institutional exclusion directly contradicts states’ obligations to provide non-discriminatory access to essential healthcare.^2^

Structural stigma refers to societal-level conditions, institutional policies, and cultural norms that restrict opportunities and reinforce the marginalisation of certain groups.^65^ In the case of abortion, stigma develops, persists, and is reproduced through laws, public attitudes, and institutional practices that are shaped by – and in turn shape – dominant societal values.^66–68^ Legal frameworks not only regulate abortion access but also signal broader moral positions, thereby legitimising stigma against those who seek, provide, or support abortion. As laws are often assumed to mirror prevailing social norms, and lawmakers are tasked with representing public opinion, understanding how the public views abortion is essential for identifying the societal contexts in which stigma is most likely to emerge and take hold. The limited number of studies that investigate the public’s attitudes towards abortion does not reflect the significant impact that public stigma deserves. Given that stigma is deeply rooted in public attitudes and tends to perpetuate itself, this area requires continued investigation. Potential interventions to reduce public stigma should be systematically tested to determine which approaches are effective at the meso level.

Our synthesis identified mitigating factors that can counteract these dynamics. Pro-choice activists actively generated counter-narratives,^56^ professional communities and unions fostered supportive spaces,^57^ and health providers framed abortion as routine medical care.^18^ These strategies illustrate that stigma reduction is possible when abortion is recognised and normalised as healthcare and when individuals can exercise their rights without fear of judgment or reprisal. Sustained interventions that address stigma at the structural level – through legal reform, inclusive media discourse, and institutional support – are therefore essential to ensure that abortion rights are not only formally granted, but also socially and practically realised.

### Conceptualisation of abortion stigma

Our review highlights the ongoing conceptual fragmentation in abortion stigma research. Many studies still depend on attribute-based definitions, especially those of Kumar et al,^5^ however, we’ve also seen a gradual shift toward multilevel and process-oriented approaches.^24,26,62^ This trend indicates a field in transition, moving away from individual-level framing toward a more structural understanding of stigma.

A key finding is the dominance of individual-level framing, even in research focusing on the general public's stigma. These approaches treat stigma as an attribute of individuals rather than a process rooted in political, legal, and institutional structures. However, as Strong^8^ argues, public stigma must be understood as structurally located, while Kumar^69^ emphasises that stigma is both a cause and a consequence of inequality. Fortunately, newer studies increasingly adopt relational and power-sensitive approaches, aligning with Millar’s^26^ view of stigma as a form of classificatory power and Love’s^70^ focus on its dynamic and shifting nature.

The continued existence of studies with vague or absent definitions highlights ongoing conceptual ambiguity. This weakens interpretive clarity and risks obscuring the roles of gender norms, religious authority, and medical hierarchies in sustaining abortion stigma. As Kumar^69^ warns, if “everything is stigma,” researchers may face analytical paralysis and become unable to identify intervention points or measure change. Our findings echo Millar’s^26^ critique of stigma’s treatment as self-evident and align with Strong^8^ and Love^70^ in advocating for explicitly structural, power-aware frameworks. Our review extends beyond Hanschmidt et al^7^ by systematically mapping definitional trends, documenting both the persistence of narrow framings and the emergence of more critical approaches. By highlighting this tension, we call for clearer, theoretically grounded definitions that emphasise the power dimensions of abortion stigma – an essential step toward improving measurement, informing policy, and guiding effective interventions.

### Strengths and limitations

The review has some limitations. We only included peer-reviewed articles, which signifies we may have missed experiences related to abortion stigma. We further limited the scope of this review to HICs, so our results may not apply to other contexts. Therefore, future research should review current studies on abortion-related stigma in low- and middle-income countries. Although we did not apply any formal language restrictions, our search terms were in English, which likely contributed to a predominance of English-language studies in our review sample, primarily from English-speaking countries. This may have limited the inclusion of relevant studies published in other languages or those conducted in non-English-speaking contexts.

A key limitation of our review concerns the extent to which existing quantitative instruments are suitable for capturing the complex dynamics of abortion stigma, particularly at the community level. A previous review^62^ evaluated abortion stigma measurements by applying the COSMIN criteria.^71^ Based on these criteria, the Stigmatising Attitudes, Beliefs, and Actions Scale (SABAS) was rated as sufficient for capturing community-level abortion stigma.^71^ However, only one study in our review^41^ employed the SABAS. Other instruments, such as the REES and the CLASS, performed less well across COSMIN standards. This highlights the need for future research to develop and promote standardised, psychometrically robust instruments that can effectively measure community-level abortion stigma across diverse contexts.

However, the strength of this review lies in its use of MMSR to systematically synthesize both quantitative and qualitative evidence, allowing us to integrate results on the effects and lived experiences associated with abortion stigma. Our research also provides a comprehensive overview of the existing research.

## Supplementary Material

Supplementary Table 2 Critical Appraisal of the qualitative studies.

Supplementary Table 3 Main characteristics of the included studies.

Supplementary Table 1 Findings of the included qualitative studies.

Supplementary Table 4. Abortion stigma definitions.

Supplementary Table 5: Overview and Categorization of Quantitative Instruments.
