# Supplementary material for: Abortion stigma amongst the public in high-income countries: a mixed-method systematic review
Source: Sex Reprod Health Matters. 2026 Feb 9;33(1):2622203. doi: 10.1080/26410397.2026.2622203 (PMC13097179; doi:10.1080/26410397.2026.2622203)
Supplement: Supplementary Table 2 Critical Appraisal of the qualitative studies. [file ZRHM_A_2622203_SM4888.docx]

Supplementary Table 2 Critical Appraisal of the qualitative studies

| **Citation** | **Q1** | **Q2** | **Q3** | **Q4** | **Q5** | **Q6** | **Q7** | **Q8** | **Q9** | **Q10** |
| --- | --- | --- | --- | --- | --- | --- | --- | --- | --- | --- |
| **Baker et al. (2023)** | N | Y | Y | Y | Y | Y | Y | Y | Y | Y |
| **Bloomer et al. (2024)** | Y | Y | Y | Y | Y | Y | Y | Y | Y | Y |
| **Baird & Millar (2019)** | N | Y | Y | Y | Y | N | N | Y | NN | Y |
| **Dianat et al. (2020)** | N | (Y) | Y | Y | Y | N | Y | Y | Y | Y |
| **Dozier et al. (2020)** | N | (Y) | Y | Y | Y | Y | Y | Y | Y | Y |
| **Duerksen & Lawson (2017)** | N | (Y) | Y | Y | Y | N | N | Y | Y | Y |
| **Duerksen & Lawson (2018)** | N | (Y) | Y | Y | Y | Y | Y | Y | Y | Y |
| **Evans & O'Brien (2015)** | N | (Y) | Y | Y | Y | N | N | Y | Y | Y |
| **Giovannelli et al. (2023)** | N | (Y) | Y | Y | Y | N | Y | Y | Y | Y |
| **Mosley et al. (2020)** | N | (Y) | Y | Y | Y | Y | Y | Y | Y | Y |
| **Sisson et al. (2017)** | N | (Y) | Y | Y | Y | N | N | Y | Y | Y |
| **Smith et al. (2016)** | N | (Y) | Y | Y | Y | N | N | Y | Y | Y |

Y= Yes, (Y) = Descriptive qualitative methodology assigned by reviewers, U = Unclear, NN= Not Needed; JBI critical appraisal checklist for qualitative research

Q1 = Is there congruity between the stated philosophical perspective and the research methodology?

Q2 = Is there congruity between the research methodology and the research question or objectives?

Q3 = Is there congruity between the research methodology and the methods used to collect data?

Q4 = Is there congruity between the research methodology and the representation and analysis of data?

Q5 = Is there congruity between the research methodology and the interpretation of results?

Q6 = Is there a statement locating the researcher culturally or theoretically?

Q7 = Is the influence of the researcher on the research, and vice-versa, addressed?

Q8 = Are participants, and their voices, adequately represented?

Q9 = Is the research ethical according to current criteria or, for recent studies, and is there evidence of ethical approval by an appropriate body?

Q10 = Do the conclusions drawn in the research report flow from the analysis, or interpretation, of the data
