# Supplementary material for: Abortion stigma amongst the public in high-income countries: a mixed-method systematic review
Source: Sex Reprod Health Matters. 2026 Feb 9;33(1):2622203. doi: 10.1080/26410397.2026.2622203 (PMC13097179; doi:10.1080/26410397.2026.2622203)
Supplement: Supplementary Table 3 Main characteristics of the included studies. [file ZRHM_A_2622203_SM4887.docx]

Supplementary Table 3 Main characteristics of the included studies

| **Study** | **Country** | **Sample size/**  **Participant characteristics** | **Goal of the study/ Phenomena of interest** | **Methodology** | **Methods for data collection and analysis (QUAL only)** | **Description of main results** |
| --- | --- | --- | --- | --- | --- | --- |
| **Bommaraju et al. (2016)** | United States, Boston & Cincinnati | Sample size: n=306  Sample description: women who had recently given birth Age: M=25.4 (SD=5.1) | The study examines the relationship between reproductive histories, race, and perceptions of abortion and miscarriage stigma among U.S. women who recently gave birth. | Quantitative approach | **/** | The study found racial differences in perceptions of reproductive stigma. White women reported higher abortion stigma, while Black and Latina women with abortion histories perceived miscarriage as more stigmatizing. These findings highlight how race and reproductive history shape stigma perceptions, emphasizing the need to consider social context in reproductive healthcare |
| **Cutler et al. (2021)** | United States | Sample size: n=886  Sample description: General Public  Age:  18–29 years: 21.7%  30–44 years: 24.9%  45–59 years: 25.4%  60+ years: 28.0% | The study aims to measure the distinct dimensions of community-level abortion stigma among U.S. adults and identify factors associated with high stigma and less favorable opinions toward abortion-supportive policies, using a nationally representative sample of both men and women. | Quantitative approach | **/** | The study found that community-level abortion stigma is prevalent in the U.S., with distinct dimensions requiring multiple tools for measurement. Average stigma levels are lower than for some other stigmatized groups but vary by political affiliation, religion, and demographics. Catholics and Republicans reported the highest stigma levels while knowing someone who had an abortion was linked to lower stigma. |
| **Cutler et al. (2022)** | United States | Sample size: n=735  Sample description: General Public Age:  18–29 years: 21.4%  30–44 years: 25.1%  45–59 years: 25.5%  60+ years: 28.0% | The study aimed to evaluate whether watching first-person abortion video stories, shared by individuals unfamiliar to participants, could reduce community-level abortion stigma among a representative sample of U.S. adults. Additionally, it sought to explore the impact of these stories on other aspects of stigma, such as warmth toward abortion scenarios and expectations of secrecy and privacy surrounding abortion experiences. | Quantitative approach | **/** | The study found that a single exposure to first-person abortion video stories did not reduce community-level abortion stigma among the general public in the long term. Factors such as the frequency of exposure, the identities of storytellers, and the specific contexts in which stories are shared might influence the impact. |
| **Hanschmidt et al. (2020)** | Germany | Sample size: n=14,459  Sample description: General Public Age:  West GER:  <18–34: 28.4%  35–49: 27.5%  50–64: 24.6%  >64: 19.3%  No response: 0.2%  East GER:  <18–34: 24.9%  35–49: 28.2%  50–64: 28.0%  >64: 18.8%  No response: 0.1% | The study aimed to analyze long-term trends in public support for restrictions on abortion access in Western and Eastern Germany after reunification, examining how social, legal, and political changes influenced attitudes. Additionally, it explored the association between abortion attitudes and barriers to abortion care to better understand their impact on women’s care pathways in reunified Germany. | Quantitative approach | **/** | The study found three distinct public attitudes towards abortion in Germany: support for unrestricted access, support only for specific cases (e.g., health risks or rape), and complete opposition. Abortion attitudes were consistently less restrictive in Eastern than Western Germany, reflecting the legacy of the former GDR’s policies. However, public support for restricting abortion access increased in both regions between 1992 and 2012. Regions with limited abortion facilities showed more restrictive attitudes, suggesting social norms influence access. |
| **Patev et al. (2019)** | United States | Sample size: n=303  Sample description: General public Age: M=36.57 (SD=12.35) | The study aims to examine the effect of abortion-stigmatizing attitudes on views regarding abortion legality while also exploring how these attitudes influence the relationship between religiosity and abortion legality opinions, controlling for political ideology. Additionally, it investigates whether gender moderates the interaction between religiosity and stigmatizing attitudes. | Quantitative approach | **/** | The study found that abortion-stigmatizing attitudes were linked to more negative views on abortion legality. For men, religiosity had a stronger influence on abortion legality attitudes when stigma levels were low, whereas for women, religiosity played a significant role at all stigma levels. Women with high religiosity and stigma exhibited more negative attitudes toward abortion legality than non-religious women. |
| **Rice et al. (2017)** | United States, Alabama | Sample size: n=642  Sample description: young adult women (18-24 years) Age: M=19.91 (SD=2.02) | The study aimed to develop and validate three scales to measure norms and stigma associated with parenting, adoption, and abortion decisions among young women. It also sought to identify social and demographic factors that influence variations in these norms and stigmas to enhance understanding of pregnancy decision-making and its impact on health and healthcare utilization. | Quantitative approach | **/** | The study developed scales to measure norms and stigma around parenting, adoption, and abortion, highlighting four dimensions: conditional acceptability, anticipated reactions, stereotypes, and attitudes. Findings showed that stigma was lowest for parenting, followed by adoption, and highest for abortion. Negative norms and stigma correlated with a lower perceived likelihood of choosing the corresponding pregnancy decision. Results emphasized that pregnancy decisions are not made in isolation but are influenced by norms around all available options. |
| **Stowers et al. (2023)** | United States | Sample size:  Sample description: adults living in the US  Age: M=38.85 (SD=11.38) | The study aimed to explore the relationship between just-world beliefs—the idea that people get what they deserve—and community-level abortion stigma in a national sample. | Quantitative approach | **/** | The study found a positive relationship between just-world beliefs and community-level abortion stigma, even after controlling for demographic factors. Stronger religious beliefs and male gender were also associated with higher abortion stigma, consistent with previous research. |
| **Baker et al. (2023)** | United States | 72 participants Age range: 18-17 years Sex: 55% women (n=30); 45% men (n=25) Race/ethicity: ​White, non-Hispanic (58%, n=32), Hispanic, ​Latino, or Spanish and multiracial Hispanic, Latino, or ​Spanish (“Latinx,” 31%, n=17), Black or African ​American and biracial Black/White (7%, n=4), biracial ​White/Asian (n=1), and biracial White/Native American/ ​American Indian (n=1)  Political affiliation: Independent (29%, n=16), ​Democrat (26%, n=14), and Republican (26%, n=14) Attitude towards abortion: Pro-life (44%, n = 24), ​pro-choice (27%, n=15), or neither pro-life nor pro-choice ​(22%, n=12). | The study aimed to gain insight into the content of people's abortion stigma and to examine the beliefs that underlie it. It explored what individuals imagine as relevant punishments for those associated with abortion care, and the logics and discourses that inform these ideas. | Descriptive qualitative | The researchers used a paper and pencil survey to collect data on participants' demographics, political attitudes, religious beliefs, and abortion identity. They categorized participants' abortion identity and religious participation levels. The researchers then used reflexive thematic analysis to inductively code the data and develop themes related to the consequences participants imagined for people who receive or provide abortion care. The themes reflected norms that abortion was imagined to violate, including legal, financial, religious, reproductive, and emotional consequences, as well as the justifications participants provided for these consequences. | The majority of participants viewed abortion as a deserved consequence or violation. Three themes emerged: (a) abortion violates the law, (b) abortion violates women's gender roles, and (c) abortion violates religious doctrine. These themes illustrate how individuals perceive abortion and justify punishing those involved, reflecting broader cultural beliefs about expected behavior and norm violations in the United States. |
| **Bloomer et al. (2024)** | Republic of Ireland and Northern Ireland | 61 trade union members | The authors examine the experiences and inequalities surrounding reproductive health issues in the workplace, with a particular focus on abortion. The research explores how abortion is discussed (or not discussed), experienced, and perceived as a workplace issue in the Republic of Ireland and Northern Ireland. | Feminist foucauldian framework | The text passages describe the methods used for data collection and analysis in a study on the positioning of abortion as a workplace issue. The researchers conducted online asynchronous focus groups with trade union members and then used Foucauldian-informed thematic analysis to examine the data. This approach allowed them to identify themes related to surveillance, normalization, and resistance, and to explore how power dynamics and socio-cultural positioning of abortion shape individual experiences and narratives in the workplace context. The analysis was a collaborative process involving the research team to reflect on assumptions and interpretations. | Workplaces in conservative societies often suppress discussion of abortion, leading to self-discipline and perpetuation of abortion stigma. Participants experienced abortion as a transgressive act against societal norms of motherhood, which limited their ability to disclose or seek help related to abortion. The normalizing gaze and disciplinary power of anti-abortion views in the workplace undermined resistance to these norms. The study provided a rare safe space for participants to address abortion stigma and consider the role of unions in challenging it. |
| **Baird & Miller (2018)** | Australia | Websites of Australia’s 36 abortion clinics & two sets of online media (Mamamia, online media of Clementine Ford) | The article examines pro-choice websites and their representations of abortion, which are often celebratory and normalizing. | Descriptive qualitative | The text examines two sites using qualitative content analysis from a feminist perspective to understand how they represent the experience of abortion and the subject position of the woman having an abortion. These themes are important in shaping the subjectivity and agency of women who have abortions and are relevant to abortion activism and legislative discussions. | The study focuses on Australian abortion clinics and pro-choice commentary, highlighting a trend towards normalizing abortion and moving away from negative representations. Clinic websites prioritize the pregnant woman's choice and emphasize lack of judgment. The emotional state following an abortion is addressed, with relief being the most common response. Overt discourse of rights is rare, but some sites warn against anti-abortion strategies. Personal narratives challenge the taboo surrounding abortion and express gratitude and relief for the decision. The study aims to reorient abortion scholarship away from negative representations and toward counter-discourses. |
| **Dianat et al. (2020)** | United States, California | 49 reproductive-aged cisgender women, Age range: 18-15, mean age of 31  Race/ethicity: Latina/ Hispanic (n=16, 33%), ​White (n=15, 31%), ​Black/African American (n=10, 20%), ​Other answers collapsed (n=7, 14%)  Education: ​Some high school, but have not graduated (n=6, 12%), ​High school graduate or GED (n=22, 45%), ​Some college or 2-year degree (n=16, 33%), ​4-year college graduate or more (n=5, 10%)  Religiosity: I try hard to carry my religious beliefs through all aspects of my life (n=23, 47%), ​My approach to life is entirely based on my religion (n=8, 16%) ​My approach to life is based on moral principles and not on the ​values of ​an organized religion (n=39, 80%) Reproductive history: Ever had sex (n=46, 94%), ​Ever talked to a clinician about birth control methods (n=44, 90%), ​Ever had a pregnancy (n=35, 71%), ​Ever had a birth experience (n=30, 61%), ​Ever had an abortion experience (n=16, 33%), ​Currently trying to prevent pregnancy (n=34 69%)  Abortion legality opinion: Abortion should be legal in all cases (n=22, 45%), Abortion should be legal in most cases (n=10, 20%) Abortion should be illegal most cases (n=10, 20%) Abortion should be illegal in all cases (n=3, 6%) | The purpose of the study was to examine reproductive-age women's attitudes toward discussing abortion during contraceptive counseling visits. The researchers also wanted to determine the best way to integrate this conversation in a way that would normalize health care discussions about abortion and improve the contraceptive decision-making process. | Descriptive qualitative | The researchers conducted semi-structured, in-depth interviews. They used a deductive-inductive directed content analysis approach to develop a codebook and coded the transcripts. They achieved inter-coder agreement and labeled response categories and themes as "dominant" or "minor" based on frequency. They selected representative quotes and organized the results by domains of inquiry, presenting relevant themes within domains and a "minor" theme with elevated attention. | The study found that mentioning abortion during contraceptive counseling can reduce stigma and help some patients make decisions, but careful communication is needed to avoid coercion. Some participants expressed concern that destigmatizing abortion too much could lead to more irresponsible behavior and more abortions. Interpersonal communication style was identified as a key factor in acceptability, with the need for nonjudgmental communication. Some participants also expressed concern about potential coercion into highly effective contraceptive methods and the emotional impact of hearing the word "abortion. |
| **Dozier et al. (2020)** | United States, Georgia | 20 Mainline and Black Protestant religious leaders  Sex: Men (80%)  Age range: 18-70+ Race: White (n=10, 50%), Black (n=10, 50%)  Marital Status: Married (n=14, 70%), Divorced (n=2, 10%) , Remarried (n=1, 5%), Single (Never Married) (n=2, 10%), Not reported (n=1, 5%)  Political affiliation: Democrat (n=13, 65%), Independent (n=4, 20%), Republican (n=3, 15%)  Tradition: Mainline Protestant (n=11, 55%), Black Protestant (n=9, 45%)  Serving in senior pastoral roles (60%) | This study seeks to understand the religious and moral views that shape religious leaders' attitudes toward abortion and their pastoral care practices, particularly in Georgia, a state with strong religious influence and gaps in reproductive health care. The study will examine the attitudes of mainline and black Protestant religious leaders toward abortion and how they provide pastoral care regarding abortion. | Descriptive qualitative | The study used semi-structured, in-depth interviews. Thematic analysis was used to identify codes and sub-themes within the data. The research team held weekly meetings to refine code definitions and discuss reflexivity in data interpretation. Patterns in the data were examined and illustrative quotes were selected for each sub-theme. | Religious leaders hold nuanced and sometimes conflicting views on abortion, with varying beliefs about when life begins and under what circumstances abortion is morally acceptable. They provide pastoral care by counseling congregants to make informed decisions, but many lack preparation and training for these conversations. Leaders emphasize empathy and compassion for those with unplanned pregnancies, but only those with pro-choice or centrist attitudes feel compelled to confront stigmatizing attitudes and behaviors toward those who experience abortion. Many leaders offer misinformation about abortion in their pastoral care. |
| **Duerksen & Lawson (2017)** | Canada | 21 participants  Sex: 8 male, 13 female Age range: 19 to 61, mean age: 25.7 years  Religion: 12 Catholic, 8 Protestant, 1 Islam  Attitude towards abortion: 10 participants reported there were no cases in which abortion was acceptable, 10 others indicated that abortion was permissible only when the pregnant woman’s health is at risk, and 1 participant reported abortion was additionally permissible in cases of rape or fetal defect | The present study examines attitudes toward women in contemporary anti-choice discourse using benevolent sexism as a guiding theoretical framework. | Realist post-positivist epistemology, theoretical framework: Benevolent sexism | Data was collected through interviews. The study used a deductive methodology and thematic analysis to analyze data on benevolent sexism. The analysis was theory-driven and based on a realist post-positivist epistemology, acknowledging the context-dependent nature of interpretation and analysis. | The study found three main themes: protective paternalism, gender differentiation, and the categorization of women. These themes are related to benevolent sexism and show that abortion is still stigmatized. Women who have abortions are seen as pitiable and poor decision makers. |
| **Duerksen & Lawson (2018)** | Canada, Victoria | 21 participants (8 male, 13 female) Mean age: 25.7 years Religion: 12 Catholic, eight Protestant, one Muslim Children: Two participants had children, 19 remaining participants 14 wanted children in the future.  Attitude toward abortion: Abortion was never acceptable (n = 10), acceptable only when the pregnant woman’s health was at risk (n = 10) and acceptable in cases of rape or fetal defect (n = 1), 15 participants expressed desire for legal restriction of abortion | This study aims to explore the views of anti-abortion individuals about abortion providers in order to explore the content of abortion provider stigma. | Social constructionist epistemology | The researcher used interviews to collect data. They used an inductive thematic analysis to examine the meanings given to abortion providers by individuals with an anti-abortion stance. They coded the data and searched for ways to combine codes into themes. The analysis was reviewed by a second author and operated from a social constructionist epistemology. The authors acknowledged that stigmatization of abortion providers is not a necessary or inherent attitude. | Participants held two types of beliefs about abortion providers: agentic and intentional actors or non-agentic victims of a larger system. The former were viewed with hostility, while the latter were viewed with compassion. Both types of beliefs are stigmatizing and can take multiple forms. |
| **Evans & O'Brien (2015)** | Australia, Queensland | Media articles concerning the Cairns abortion case (n=150), print and online media articles, the majority of the articles (n=99) were published by the Cairns Post, Australian Associated Press, Sydney Morning Herald, ABC Online and The Australian. | This article is based on research analyzing 150 media articles about the Cairns abortion case to determine how language and terminology choices can contribute to constructions of deviance. | Descriptive qualitative | Articles on abortion were collected through internet search engines and online databases. They were analyzed using coding and deviance theory. The coding involved counting key terms and identifying pro-life, pro-choice, or neutral stances. The analysis focused on how language stigmatizes abortion and constructs those who abort as deviant. The article does not provide a detailed quantitative analysis but examines the emergence of key themes in language and media discourse. | The article examines media language in the Cairns trial and identifies themes that contribute to the stigma surrounding abortion, including the humanization of the fetus, the stereotyping of mothers, and the labeling of women who abort as "depraved. Language that focuses on the method of abortion creates another level of deviance. Despite popular support for decriminalization, pro-life language still shapes perceptions. The Cairns case highlighted the stigmatization of women who choose abortion, and changes in the law have been minimal. |
| **Giovannelli et al. (2022)** | Italy | 34 cisgender female pro-choice activists  Mage: 44.41 Additional: Majority of ​the participants came from the center of Italy (58.8%), had acquired higher education ​(73.5%), were atheists (70.6%), and had a left-wing political orientation (94.1%) | The study aimed to deepen the understanding of these activists' subjective experiences, opinions, and feelings about the derogatory actions, stereotypes, and prejudices they face, as well as the strategies they use to combat their own stigmatization and abortion stigma in general. | Descriptive qualitative | The researchers conducted semi-structured interviews either in person, via telephone, or via Skype. The audio recordings were transcribed and analyzed using a qualitative directed content analysis method, focusing on the key concepts of experienced, perceived, and internalized stigma. The researchers developed a codebook, with each interviewer coding the transcripts individually before discussing and resolving any discrepancies. The findings were translated into English, and pseudonyms were used to protect the participants' identities. | Participants perceive themselves as targets of negative stereotypes and behaviors but do not internalize the stigma. They use a variety of strategies to cope, including being open about their activism. Key findings reveal themes of experienced, perceived, and internalized stigma, as well as strategies participants use to cope with stigma. In addition to adopting stigma management strategies, participants also report using them to deconstruct stereotypes and prejudices about elective abortion by emphasizing self-determination, freedom, and critical engagement with key concepts used in anti-choice rhetoric. |
| **Mosley et al. (2022)** | United States, Georgia | 20 Religious leaders, Senior Pastors, Youth Pastors, and lay leaders (i.e., non-clergy congregants chosen for leadership roles within the church) | The researchers examined differences between white and black Protestant attitudes toward these groups, church practices regarding them, and implications for church-based health programs. | Descriptive qualitative, multi-level conceptual model that primarily, theories: theory of Triadic Infuence (Flay et al., 2009) (intrapersonal, social situation, and culture), Moral Foundations Theory (Graham et al., 2013), and stigma (Hatzenbuehler et al., 2013) and intersectionality (Crenshaw, 1989; Mullings & Schulz, 2006) frameworks | The research team conducted in-depth interviews with religious leaders, using a codebook developed through iterative team-based reviewing and memo-ing. Thematic analysis was used to describe the leaders' attitudes and beliefs. Themes were developed using techniques such as memo-ing, group comparisons, code matrices, and diagramming. | The study found that religious leaders held different attitudes toward abortion and sexuality, with some adhering to traditional judgments based on religious scriptures and constructs of sexual purity, while others emphasized love and inclusiveness for all individuals. Tensions existed between these two perspectives. Some participants provided examples of how to build supportive and empathetic norms, including LGBTQ inclusivity, through personal relationships and de-stigmatizing witness. |
| **Sissona et al. (2017)** | United States | 31 staff writers (n=20) and freelance reporters (n=11) Age range: 21-70 | The authors sought to explore how journalists understand their role and experience in covering abortion today. | Descriptive qualitative | The researchers conducted in-depth interviews and used shared notes to identify emergent themes and develop a preliminary code list. They audio-recorded, transcribed, and analyzed the interviews using modified grounded theory techniques. The iterative process of coding and analysis allowed for identification of new themes and challenges. The second author coded all transcripts, and the first author reviewed them to determine when analytical saturation was reached. | Journalists face challenges in covering abortion, including maintaining neutrality, finding new angles, and facing harassment from anti-abortion activists. They also experience and can create stigma in their reporting. Stigma can lead to distancing from the issue, incorporation of misinformation, difficulty finding sources, and a decrease in the quality and relevance of abortion journalism. |
| **Smith et al. (2016)** | United States, Alabama | Six focus groups with 34 participants and 12 cognitive interviews (n=46) with low-income women Age range: 19-24 Race/ethicity: black (52%), 41% white, and 7% ​​identified as other  Prior pregnancies: 54.4% | The study explored perceptions of norms and stigmas related to unintended pregnancy, parenting, adoption, and abortion, and to examine racial differences in these perceptions among young, low-income women. | Descriptive qualitative | The researchers used a one-time focus group and developed codes based on previous research themes. They analyzed perceptions of behavior, attitudes, and blame regarding stigmatized behavior. The first and last authors coded initial transcripts and developed a revised codebook. The first author analyzed all remaining transcripts. Preliminary findings were presented to the research team and colleagues for quality control. | The community expects pregnancy to occur in monogamous relationships with mature, educated, and financially stable partners. However, unintended pregnancy outside of these circumstances is common, and young women are expected to bear and raise their child. Women who choose to do so are viewed more positively than those who choose abortion or adoption. |
