# Supplementary material for: Abortion stigma amongst the public in high-income countries: a mixed-method systematic review
Source: Sex Reprod Health Matters. 2026 Feb 9;33(1):2622203. doi: 10.1080/26410397.2026.2622203 (PMC13097179; doi:10.1080/26410397.2026.2622203)
Supplement: Supplementary Table 1 Findings of the included qualitative studies. [file ZRHM_A_2622203_SM4886.docx]

Supplementary Table 1 Findings of the included qualitative studies.

| **Study: Baker *et al.*** [30] **Abortion Stigma: Imagined Consequences for People Seeking Abortion Care in the United States** | |  |
| --- | --- | --- |
| Finding | “Murder” and Legal Punishment. When describing legal consequences, participants drew upon discourses of “abortion is murder” when describing the consequences they wanted to see directed toward those associated with abortion. (U) |  |
| Illustration | “Descriptions of abortion included references to “manslaughter,” “murder,” “homicide,” and “killing” when discussing abortion and “the baby” and “the child” when referring to the fetus.” ^(p.40)^  “There should be legal consequences. Because [abortion] is murder. That is to say, there are legal consequences because it is murder, you are killing a creature even though it is your child. A lot of things come to mind, to say there should be fines, but also a punishment, jail, because it is a murder. For life, for murder, that is why these people should be put in jail.” ^(p.40)^ |  |
| Finding | The legal consequences of “murder” were most often applied to women, but were sometimes applied to abortion care providers. (U) |  |
| Illustration | “I like what Alabama or Georgia, I think, set up…a system where doctors who do it would be punished, and I think that’s the right way to do it… make it the same as the rest of your penal system for homicide” ^(p.40)^ |  |
| Finding | Analogies to Crime. In addition to incarceration, participants used analogies with crimes and legal terms to justify other types of legal punishments, such as fines and probationary periods. (U) |  |
| Illustration | “For example, Participant C called for a “ticket” like “when you’re stopped by traffic police” ^(p.40)^ |  |
| Finding | Descriptions characterized women as criminals with accomplices: (U) |  |
| Illustration | “having one abortion was described as a “first offense” (Participant D, woman, age 45, pro-life, White, mod relig.) and her sexual partner was “the partner to the crime” (Participant E, man, age 65, pro-choice, White, mod relig.).” ^(p.40)^ |  |
| Finding | Participants’ willingness to punish law violators illustrated the power they gave to the law to determine right from wrong, enforce stigma, and justify punishment. (U) |  |
| Illustration | “I guess it just depends on the law at that time and whether it’s legal or not at that time, 40 Psychology of Women Quarterly 47(1) because it’s gone back and forth” ^(p.41)^ |  |
| Finding | Controlling Reproduction. In this theme, descriptions included forcing women to use contraceptives or be sterilized (without their consent). […] These linkages between sterilization and abortion—either forced or assumed to be an inevitable consequence—demonstrate how women and their reproductive capacity were imagined to be threatened by seeking abortion care. (U) |  |
| Illustration | “Participants characterized women seeking abortion as having made “bad decisions” and needing to “get fixed.” ^(p.41)^  “Sterilization, as an extreme… if they made a bad decision and they just don’t want the kid, I think that they need to be sterilized” ^(p.41)^ |  |
| Finding | Forced or coerced contraceptive use and sterilization are human rights abuses, and the desire to punish women who have abortions using these methods illustrated the severity of abortion stigma. (U) |  |
| Illustration | “You’re pregnant again and now you want another abortion?…The consequences are: we’re not going to do this again, we’re going to put you on birth control that’s going to work or we’re going to get you sterilized” ^(p.41)^ |  |
| Finding | Along similar lines, participants imagined that accidental sterilization could occur as a result of abortion. (U) |  |
| Illustration | “This piece of misinformation was repeated as truth, characterized as unfortunate, and considered something that participants “wouldn’t wish on anybody” ^(p.41)^ |  |
| Finding | Imagining Women as Emotional. We found a persistent expectation that women would feel emotional consequences following an abortion. (U) |  |
| Illustration | “These included guilt, regret, resentment, posttraumatic stress disorder, depression, and a “weight on her conscience.” Participants described these feelings as “serious,” “deep,” “major,” “a lot,” “a weight,” and as lifelong.” ^(p.41)^ |  |
| Finding | Participants felt certain that these feelings would occur, invoking norms and expectations surrounding women’s emotions and their innate bond with the aborted fetus. (U) |  |
| Illustration | “Naturally the woman will suffer consequences just by having [the abortion] done. The mom is going to have pain from it, you know, mental, emotional” ^(p.41)^ |  |
| Finding | Descriptions in this theme endorsed a narrative that negative feelings after an abortion are inevitable, long-lasting, and even debilitating and requiring the aid of state-mandated counseling. (U) |  |
| Illustration | “For example, Participant J said that women who have abortions “maybe should go to counseling… maybe joining a support group for women that have had abortions” ^(p.41)^ |  |
| Finding | More directly connecting abortion to a gender role violation, (U) |  |
| Illustration | “Participant K stated women who have abortions should go to therapy because “even legal abortion has serious emotional and physical trauma on a woman, and they would need help to get over all the emotions that go with getting rid of your child” ^(p.42)^ |  |
| Finding | These responses reflected Shellenberg and Tsui’s (2012) findings that shame from others is related to women’s sense of shame or guilt over abortion. (U) |  |
| Illustration | “I think in most cases, anybody who has an abortion either knows somebody or is part of a community or they, themselves, are conflicted morally about it” ^(p.42)^ |  |
| Finding | Community-level shame was not critiqued, however, and was even supported in some instances. Participant M argued in favor of social punishment for revealing an abortion procedure: (U) |  |
| Illustration | “If you were to do something like that [get an abortion and] if your family finds out and then they think differently of you, I mean, you deserved that. You put yourself in that situation” ^(p.42)^ |  |
| Finding | Punishment and Religious Justifications. This theme illustrates how abortion was imagined to violate religious tenets, sometimes imagined as such a severe violation that a person ought to be isolated from their religious community. (U) |  |
| Illustration | “Participant O argued that women who have abortions “just get paid back by the Lord” (man, age 49, neither abortion identity, White, high relig.), indicating his certainty in the retributivism of his God.” ^(p.42)^ |  |
| Finding | Some pointed to their religious values when imagining the consequences of abortion. (U) |  |
| Illustration | “Participant P drew on Catholic and Evangelical anti-abortion doctrine when he said that “every person, whether a baby, a heartbeat, is made in the image of God” ^(p.42)^ |  |
| Finding | Implied Religious Punishment. Other participants were less certain about whether and how a divine power would pass judgment on people involved in abortion care. (U) |  |
| Illustration | “The Lord’s going to have the end all decision; if there were to be a consequence, He’ll take care of it” ^(p.42)^  “Karma plays its role in this, and I think karma would be the verdict in the situation” ^(p.42)^ |  |
| Finding | State Punishment of Gender Role Violators. With regard to violating gendered expectations, women who were identified as legally punishable were those who transgressed their role as caregivers by prioritizing their careers or financial health over motherhood. (U) |  |
| Illustration | “If someone was having an abortion just strictly to maintain their lifestyle and if a life is taken for financial gain, then maybe financial consequences would be appropriate… The inconvenience of [a child] maybe interfering with career or something like that or just being unwilling to pay the costs of a child.” ^(p.43)^ |  |
| Finding | State Punishment of Low-Income Women. Low-income women were explicitly targeted in participants’ descriptions and individuals often drew on images of State intervention or approval. (U) |  |
| Illustration | “When I think of [legal consequences], I think [of] the lower income people. Take away the food stamps, [laughs] you know? I mean, don’t give them government assistance” ^(p.43)^ |  |
| Finding | Similarly, Participant U suggested forcing low-income women to use contraceptives (U): |  |
| Illustration | “Maybe they should be forced [onto] birth control or something for a period of time if you are unable to pay for the abortion yourself” ^(p.43)^ |  |
| Finding | These remarks framed low-income women as uniquely deserving of surveillance and punishment. (U) |  |
| Illustration | “The government should be able to say, ‘You’re done [getting pregnant]’” ^(p.43)^ |  |
| Finding | State Punishment of Religious Violators. In addition to gendered norms, participants also described how they desired the law to reinforce and uphold religious tenets and gendered norms simultaneously. (U) |  |
| Illustration | “I don’t think you should ever get out of [prison] because you killed… When you go to jail, there are Bibles there, and that’s when you stop to think, to meditate and start to see what is bad, the wrongdoing, right?” ^(p.43)^  “Participant X similarly suggested that women who have abortions “should be found out” and forced to “rethink and revisit” their religious beliefs” ^(p.43)^ |  |
| Finding | Participant Z said that she only heard ideas about punishing women in the news, but when asked to expand on the consequences she heard about, she instead emphasized a pro-choice position: (U) |  |
| Illustration | “Mostly, I hear that it should be the woman’s choice. I had a roommate that had an abortion…I took her and picked her up” ^(p.43)^ |  |
| Finding | Participants justified not desiring consequences for abortion through implicit and explicit references to their abortion attitudes and discussion of the broader implications of punishment. (U) |  |
| Illustration | “No, I don’t think so because that’s not fair. I don’t feel like anybody should get hurt” ^(p.43)^  “I just don’t think that there’s anything wrong with getting an abortion, so having a consequence for something that I don’t feel is wrong doesn’t make sense” ^(p.43)^ |  |
| Finding | However, individual attitudes toward abortion were not the only determinant in endorsing consequences for abortion. (U) |  |
| Illustration | “If women want to have an abortion, they’re going to find a way to have it, and then they could go to more outlandish [laughs], you know, resolutions, I guess, instead of looking for actual doctors who know what they’re doing.” ^(p.44)^ |  |
| **Bloomer *et al.*** [38] **The workplace as a site of abortion surveillance.** | |  |
| Finding | Participants identified how abortion stigma was a key component of self-discipline, preventing women from raising the issue of abortion in the workplace, and a major barrier to engaging with employers. (U) |  |
| Illustration | “I worked with a colleague who made the decision to have an abortion … they did not disclose this  information to our employer as they were so worried about the stigma. Instead my colleague took sick leave to travel to England for their procedure … the associated stigma with abortion means very few people would go to an employer and tell them that's why they need time off. This then adds to the pressure of an already stressful situation. Lying to your employer, taking sick leave, having to make travel arrangements while worrying if you get 'caught' how will you explain yourself. Then the financial implications are another added pressure.” ^(p.9)^ |  |
| Finding | Participant P22-Y confirmed the disciplinary power of abortion stigma when considering her experience of supporting a colleague who took annual leave when she traveled to England for an abortion, suggesting that (U) |  |
| Illustration | “there's no way she would have told her supervisor why [she took leave] … she just said she was visiting relatives in England”. ^(p.9-10)^ |  |
| Finding | Very similar negative experiences in the workplace were reported, with pregnant women typically restricting their behavior to confiding only in one colleague. In this example the person confided in reflects on the support that might have been offered from colleagues. (U) |  |
| Illustration | “As I said, the employer didn't know that she had an abortion, however, she took time off to go to England and she was acting so differently when she came back, that I've since wondered if anyone guessed. Nobody said anything to her as far as I know, but I suppose I wish someone could have helped her.” ^(p.10)^ |  |
| Finding | Participants recognized that the impact of abortion stigma in the workplace was a significant factor, in that it (U) |  |
| Illustration | “leaves the voice of the thousands of women who have been affected by poor access to abortion, out of the discussion” ^(p.10)^ |  |
| Finding | Stigma, allied to the threat of criminal punishment, which was present at the time of the study in 2017, combined with a lack of support, results in many women who have experience of abortion shying away from raising abortion as a workplace issue. (U) |  |
| Illustration | “That the abortion experience remains a private and personal matter was confirmed by P55, reaffirming that “maybe women don't want to disclose that they have had the procedure done, probably because there is still so much stigma surrounding abortion in Ireland”, an impact of silencing magnified for women in male-dominated settings.” ^(p.10)^  “I work in a male dominated work place and have noticed that they seem afraid to discuss abortion and don't like when it is brought up.Maybe unions getting involved would open up the conversation more in the work place, because the stigma definitely needs to be challenged.” ^(p.10)^ |  |
| Finding | Aligned to this was the observation that the opportunity to discuss abortion as a workplace issue with fellow trade unionists in the focus groups was welcomed as an antidote to the secrecy demanded by abortion stigma. (U) |  |
| Illustration | “Following the focus group activity, some participants modified their perspectives on the issue, with open discussions viewed as a key component in “normalizing and de-stigmatizing abortion” (P13-Y) and in broadening the remit of the trade union movement.” ^(p.11)^  “I found the forum useful in the sense of clarifying for myself as a trade unionist what I think the correct political position of the union ought to be in this complex issue. Until the forum I had not considered abortion as a workplace matter. However now I do see it as a workplace matter that deserves union acknowledgment.” ^(p.11)^ |  |
| Finding | Here we also observe the framing of the fetus referred to as “child” and “baby” and the act of abortion as “murder”. (U) |  |
| Illustration | “I'm also not judgemental… I honestly believe though that abortion is being pushed to be normalised in society lately—women's right over her own body etc… As a young woman myself I agree with the principal of women having rights over your own body. What I disagree with is when it's at the cost of murdering a child who can't protect itself … Having a baby bump, feeling the baby kick, just a few weeks off delivering and suddenly deciding you don't want the baby anymore so you will kill it—I just don't understand that.” ^(p.11)^ |  |
| Finding | The anti-abortion position amongst participants traversed a continuum that included those who would deny abortion in all circumstances, wherein any relaxation of the abortion legislation as likely to lead to a “slippery slope” of abortion on demand (P56–Male; P23–Male; P7-Male; P24–Male). (U) |  |
| Illustration | “Outright opponents of abortion positioned abortion as “murder”: “murdering a child who can't protect itself” ^(p.12)^  “So you [the researchers] are asking for views of "normalisation" of abortion. The question itself appears biased as you thinking abortion is "normal". It is not normal to murder defenceless individuals because it is "trendy" ^(p.12)^ |  |
| Finding | The view of abortion as a lifestyle choice is common in abortion mythology and presents women who have abortions as selfish and typically young and reckless. In this mythology, pregnancy is a punishment for sexual behavior. (U) |  |
| Illustration | “The absolutist viewpoint characterized abortion clinics as “human abattoirs” ^(p.12)^  “Any relaxing of legislative restrictions would begin “normalizing abortion as a form of contraception”, the demand for which is driven not by reproductive rights but “to protect lifestyles, imagine sacrificing a baby's life to protect a lifestyle” ^(p.12)^ |  |
| **Study: Braid and Millar*.*** [35] **More than stigma: Interrogating counter narratives of abortion** | | |
| Finding | Clinic websites uniformly place the pregnant woman who is considering or seeking an abortion at the centre of the imagined world of the clinic […]. (U) |  |
| Illustration | “We will support your choice’ (Options) and ‘[t]he right procedure for you depends on your preference.” ^(p.5)^ |  |
| Finding | Some sites explicitly reiterate the key feminist principle (Albury, 1999: 50) that: (U) |  |
| Illustration | “someone else should not make the decision for you. You know best about your own life” ^(p.5)^ |  |
| Finding | No reason for having an abortion is privileged or discounted and, how-ever the decision-making process is represented, all abortion providers privilege choice, or ‘options’, as the key value: (U) |  |
| Illustration | “Our Philosophy of Patient Care is to provide women with choice” ^(p.6)^ |  |
| Finding | Nearly all the websites address the post-abortion emotional state. (U) |  |
| Illustration | “Usually, psychological and emotional well-being improves after an abortion—and it’s rare to experience long-term negative consequences” ^(p.6)^  “evidence shows that there are rarely lasting negative consequences for women who choose to have an abortion’” ^(p.6)^  “About 90% of women feel relieved following an elective abortion, and engage positively with their lives” ^(p.6)^ |  |
| Finding | Another site invokes abortion stigma in order to challenge it by normalising abortion as a medical procedure: (U) |  |
| Illustration | “‘Our philosophy is to remove that stigma, by making the procedure as acceptable and respected as any other gynaecological operation.” ^(p.7)^ |  |
| Finding | Several sites warn about ‘deceptive ‘‘counselling’’ services’ (GCA; Greenslopes; RWH), a reference to anti-abortion ‘false providers’ (Allanson, 2007) that advertise counselling services without declaring their opposition to abortion. (U) |  |
| Illustration | “Some people, especially those who are opposed to abortion, believe that most women sink into a pit of depression, guilt and grief after an abortion. This is false” ^(p.7)^ |  |
| **Study: Dianat *et al.*** [31] **Breaking the silence in the primary care office: patients' attitudes toward discussing abortion during contraceptive counseling** | | |
| Finding | Another uniquely helpful quality noted about Example 1 was its destigmatization of unplanned pregnancy and abortion. (U) |  |
| Illustration | “… it's helpful because say that does happen, you have a mistake. […] you already know that your doctor is understanding that things do happen and […] he or she already has discussed with you your three options. So, […] that is a little bit more approachable […] – because it's scary if you do have a unplanned pregnancy. So, maybe just having this already in your ear, […] you could go back and let them know, you know, a mistake did happen, and I want to now talk about my plan with my pregnancy.” ^(p.3)^ |  |
| Finding | Abortion as a back up method. Unhelpful. Destigmatizing. (U) |  |
| Illustration | “I don't think a doctor would even have to bring that up.People know, you know, what they're doing. I mean, people know that you can either be a parent, or abort the child, or - like give the child up for adoption. […] I think if the doctors continue to say that to people, then […] people would just be more irresponsible. […].” ^(p.3)^ |  |
| Finding | Mention of abortion to assess acceptability of pregnancy management options. Centers Pregnancy. (U) |  |
| Illustration | “It just put a bad stigma on the whole overall thing, you know, birth control, sex, pregnancy… I wouldn't even want to talk about birth control. […] because she just scared the shit out of me. Like, let's spring your question around you gettin’ pregnant in the next few - I don't think some doctors  understand how hard it is for a woman to even open up about things about birth control, pregnancy, terminating pregnancies, and what their options is.” ^(p.3)^ |  |
| Finding | Mention of abortion to assess acceptability of pregnancy management options. Stigmatizing. (U) |  |
| Illustration | “I would definitely be thrown off because I'm coming to my provider for options to prevent, not to be questioned. […] they're not bad questions, but I would definitely […] already feel like he is ready to judge.” ^(p.3)^ |  |
| Finding | Those who thought this was helpful believed that mentioning abortion during contraceptive counseling would allow patients to ask questions and seek care more comfortably. (U) |  |
| Illustration | “I guess, I would be like, ‘Oh, yea, I guess that is a form of birth control.’ Like it would just remind me that it's out there. You know what I mean? ‘Cause when I'm thinking of birth control, I'm not thinking about abortion. I'm thinking, you know, the other things […it's helpful that] it's listed as an option…” ^(p.3)^ |  |
| Finding | Further among those who found the integration of topics acceptable, when asked to consider what could be potentially unhelpful about this, some said that mentioning abortion during contraceptive counseling could destigmatize it “too much.” (U) |  |
| Illustration | “…that's like giving somebody a free pass… I've seen it for myself, like, I don't take these pills, we don't use condoms, if I get pregnant, I can just go to the clinic and… you know what I mean?” ^(p.3)^  “Um, it seems like it shouldn't be there. […] not even thinking about the moral implications—[abortion] as a birth control method is the most invasive and, like, as a health care provider, […] suggesting an option that is the most invasive seems like the wrong way to go about it.” ^(p.3)^ |  |
| **Study: Dozier *et al.*** [32] **Abortion attitudes, religious and moral beliefs, and pastoral care among Protestant religious leaders in Georgia** | | |
| Finding | Pro-life. Those with “pro-life” attitudes felt abortion is “too common” and “ought to be a last resort” that is not rushed into or taken lightly given the gravity of its implications. (U) |  |
| Illustration | “Pro-life but not in a Republican religious right type of way . . . pro-life for the baby . . . for the mother, and for people when it comes to healthcare.” ^(p.7)^  “I’m not for abortion . . . you don’t give a woman the right to make the decision whether or not to kill when she’s pregnant.” ^(p.7)^ |  |
| Finding | Some participants were less decided about the moral acceptability of abortion in cases of rape, stating tensions between the belief that something good might come out of the pregnancy and concerns for the mental health of the mother. (U) |  |
| Illustration | "One Mainline Protestant pastor raised concerns about abortion in cases of rape and expressed that it may be morally acceptable only when the woman has no shared “responsibility for [the pregnancy],” such as a woman being under the influence of alcohol versus being “attacked by an evil person.” ^(p.8)^  “The same participant explained that abortion is not a morally acceptable option for fetal anomaly because such anomalies are the result of “the sinful nature that we’re born into (due to original sin of Adam and Eve)”, and because God does not make mistakes, therefore all pregnancies should be carried to term.” ^(p.8)^ |  |
| Finding | Attitudes in the “gray area”. Participants cited tension between belief in the sanctity of life and respect for individual autonomy. (U) |  |
| Illustration | “I tend to be more conservative on this issue. I’m not blockading abortion clinics, but I’m not out waving the banner of pro-choice either. So I guess I’m somewhere in the middle. But I would hold to the sanctity of life, and share that with people, without a doubt.” ^(p.7)^  “That’s my tension. It’s like I’m not for it at all, but (God) gives me free choice every day. So that’s my dilemma. Now if you were to ask me what I vote for, pro-life, pro-choice, I’m definitely pro-life. But I still—that’s the tension a little bit. All right? ’Cause it doesn’t stop.” ^(p.7)^  “[The church’s] decision probably has always been pro-choice. BUT we advocate life.” ^(p.7)^  “'So I don't believe that it's my right as a human being to tell a woman what they're going to do with their body because a woman and a child are literally inexorably linked as far as being in utero and in womb. And so I have no right to say to someone who's carrying a child that you can or cannot do this or that or the other because that child is your body and you have the right to see your body. But at the same time, there is also the potential for a life being carried inside of that body. And the part of me that values the sacredness of all life says, ‘Oh, but look at the potential there. Look what good that future human being could do in the world.’ So I sort of stand at a very strange gray area and crossroads with abortion.” ^(p.8)^ |  |
| Finding | Some participants in the “Gray Area” cited that abortion may be the best decision for some people if having an abortion would alleviate potential suffering or in cases in which a mother is not able to care for herself or for a child. (U) |  |
| Illustration | “My personal views on abortion is that I believe that in some cases, abortion could be the best option for that individual, if they come to that conclusion, such as poorly–development of a fetus that does not have, medically speaking, the chance for–productive or normal life outside of the womb. Okay? People make those decisions on their own. One of the more sensitive issues is having a healthy child, but if that child is the result of rape or incest, I don't believe that God cannot forgive anyone for any decision that possibly could be against his will. His will, of course, is that we have life, but I also hold to this belief that every, every, every–conceivable sin is forgivable by God, except for blasphemy . . .” ^(p.8)^ |  |
| Finding | Pro-choice. These participants discussed tensions between the need for abortion and the need for women to exercise bodily autonomy. (U) |  |
| Illustration | “I’m a firm believer that it’s a woman’s right to choose. It’s your body . . . and it’s your life. . . . I love adoption. I think that’s a great option, but it’s an option. . . . I don’t know every person’s personal story, what they’ve gone through, what they have to go through. So, I don’t know that it has anything to do with me, so why should I have any determination or even any philosophy or theology on it?” ^(p.7)^  “We have a very conservative (denomination). For the most part, we’ll use the word progressive, they’re on the progressive scale. I mean, gay marriage–we changed our canons to allow for gay marriage. We’re not against contraception. We are–we consider ourselves pro-choice. When we say it, we mean pro-family, pro-child, and life begins, and life doesn’t end until a person dies, not by the hands of the state.” ^(p.7)^ |  |
| Finding | They felt pregnancy-related decision making should rest with a pregnant person and God, but they would try to guide people considering abortion to the best outcome for the mother and the baby. They emphasized that their pastoral care would consist of much listening and understanding. (U) |  |
| Illustration | A senior pastor at an Episcopal church who identified as “pro-choice” explained that he could not make decisions about abortion for people because to do so would be “treading on a violation of the relationship between [them] and God.” ^(p.9)^ |  |
| Finding | Redemption. Many participants across the attitude spectrum expressed that there is a process of healing, redemption, or becoming “whole” that women must undergo following an abortion to resolve adverse psychological and spiritual effects. Most participants describe these effects, such as emotional guilt, regret, and spiritual effects, such as questioning whether God would forgive them. (U) |  |
| Illustration | “They expressed that these effects are often lasting and lifelong for those who cannot or will not “do the work [of] resolving their own minds.” ^(p.10)^ |  |
| Finding | Many participants across the spectrum of attitudes expressed the importance of not condemning a person because of abortion, citing scripture punitively, or passing judgment. A participant from an Episcopal church who identified as “pro-choice” said, (U) |  |
| Illustration | “. . .I also think it’s letting them know that they’re loved. I go back to that, with–that’s–love will win. I know it’s become sort of a moniker and nobody takes it seriously, but– . . .It will. So how do we love? How do we love that woman who didn’t plan, and that baby that’s going to result from it? Or the woman that planned, and still she got pregnant. It’s not what do we with them. It’s how do we love them, and make sure they know they’re loved? That’s the part I see my role as.” ^(p.12)^ |  |
| **Study: Duerksen and Lawson*.*** [36] **Not Brain-washed, but Heart-washed: A Qualitative Analysis of Benevolent Sexism in the Anti-Choice Stance** | | |
| Finding | Rachel expressed sadness that abortion is often cloaked in shame and secrecy (U) |  |
| Illustration | “They feel ashamed that they shouldn’t have done it or they shouldn’t have gotten pregnant and all these things… it breaks my heart” ^(p.866)^ |  |
| Finding | Wendy expressed a concern that the pro-choice movement and efforts to reduce negative stigma surrounding abortion could actually be increasing shame and secrecy (U) |  |
| Illustration | "I think especially recently with that whole #Shout your Abortion hashtag… the voices of the women who’ve had negative experiences with abortion or regret their abortions, like their voices are being put down or are being silenced"^(p.866)^ |  |
| **Study: Duerksen and Lawson.** [37] **‘Removed from humanity’: a qualitative analysis of attitudes toward abortion providers in anti-abortion individuals in Canada** | | |
| Finding | Many participants felt abortion providers could be ignorant individuals who do not recognise the harm they cause. (U) |  |
| Illustration | "Individuals discussed the possibility that providers ‘could feel like they did the woman a favour’ (Henry) or ‘could feel good about providing women freedom’ (Gloria). ^(p.454)^ |  |
| Finding | However, these well-intentioned providers were ultimately seen as ignorant: (U) |  |
| Illustration | “They probably feel like they’re helping a lot of women. You know, like each abortion is a woman they’ve saved from crisis … I can’t judge them for their ignorance. I’m glad they want to help women. I wish they knew more about what they were actually doing.” ^(p.454)^ |  |
| Finding | Providers were believed to be ignorant of the effects of abortion on women, as Penelope  recommended providers (C) |  |
| Illustration | “hang out with these women before, during, and after the abortion … just to see what is actually happening at their psychological and physiological level” ^(p.454)^ |  |
| Finding | Abortion providers were also seen to be ignorant about conception and fetal development: (U) |  |
| Illustration | “[It’s] scary that med school didn’t teach [abortion providers] that conception creates a unique … set of DNA that has all the information necessary … to make a baby nine months later. That’s scary to me that there are doctors who don’t know that or don’t think that, because that’s just scientific” ^(p.454)^ |  |
| Finding | A few participants also discussed that abortion could be a traumatising experience for the provider, stating that providers ‘could feel like… (U) |  |
| Illustration | “wow, I just murdered someone” ^(p.455)^ |  |
| Finding | Wanda stated that performing an abortion traumatises providers in a way that other medical procedures do not by citing personal experiences she read of one abortion provider: (U) |  |
| Illustration | “He basically said that they have nightmares about what they’ve had to do and they dream that they’re pregnant and having abortions, or about babies that they’ve had to dismember … this is very revealing, because you don’t see this kind of thing with any other medical procedure. Nobody has nightmares about performing wisdom tooth extractions or open-heart surgery.” ^(p.455)^ |  |
| **Study: Evans and O’Brien.** [34] **The Cairns abortion trial: Language, deviance, and the ‘spoiled identity’** | | |
| Finding | By referring to the foetus as a baby and referring to the damaging effect of abortion on the foetus, the language is constructing this image of murder and therefore stigmatising those who choose abortion as murderers. (U) |  |
| Illustration | "What about the baby – it certainly suffered an “ill effect”!” ^(p.759)^ |  |
| Finding | The analysis of the news articles discovered that language and key terms are evident in abortion discussions which demonise and isolate women who obtain an abortion (U) |  |
| Illustration | "‘We don’t believe that abortion ever helps a situation, it does harm, it harms physically, mentally, spiritually and emotionally” ^(p.760-61)^ |  |
| Finding | This language stigmatises the procedure of abortion as leaving women ‘wounded’, with their body now ‘a place of death’ which causes harm ‘physically, mentally, spiritually and emotionally’ (U) |  |
| Illustration | "good-hearted women whose inner lives have been wounded by abortion – having created a place of death in their body" ^(p.760-61)^ |  |
| **Study: Giovannelli *et al.*** [40] **Fighting for abortion rights: Strategies aimed at managing stigma in a group of Italian pro-choice activists.** | |  |
| Finding | Experienced stigma. Participants’ narratives revealed that the cisgender female pro-choice activists we involved in the study were targets of verbal and physical mistreatments as a result of their pro-choice beliefs and roles in pro-choice movements and organizations. (U) |  |
| Illustration | “Several participants said that they had experienced (or witnessed) verbal attacks in person or online, including being called murderers, sluts, stupid, and witches, with the intent of devaluing and denigrating them, especially by no-choice people or right-wing people.” ^(p.7)^ |  |
| Finding | While many of the reported episodes occurred during public demonstrations in support of abortion, others happened during informal debates between acquaintances and within the family, as well as after posting pro-choice comments or news on social networks. (U) |  |
| Illustration | “[People] called us “killer of innocent victims”, a word that people used in the past and now […]. According to people, we kill children […].”^(p.7)^  “It happened [verbal aggression] to me in several demonstrations. I remember an episode … I remember they shouted at me insults such as “whore” and so on. […] Another time, I was with other activists and we had banners in our hands. I remember that the people who manage the security service hurled at us grave offenses. […] It happened that they shouted to us, “go home, go home, your place is at home”. These insults were very common: one of us came out of a bar and people shouted at her.” ^(p.7)^ |  |
| Finding | Several participants had received (or read) insults with offensive images that depicted a hyper--humanized embryo/fetus to warn them that the embryo/fetus is a human being from conception. (U) |  |
| Illustration | We were constantly insulted on Facebook. On our Facebook page, there are people who called us bullies because we denounced a pharmacy. This pharmacy was defined by the owner as a “conscientious objector pharmacy”. In Italy, pharmacies cannot object; he [the pharmacist] gave the wrong information. After we published this denouncement, we received the worst insults. We were called “murderers”; people wrote to us: “we want to see if your mother had an abortion” […]. Some people published dubious scientific evidence about abortion on our Facebook posts and they sent us pictures of fetuses […].” ^(p.8)^ |  |
| Finding | A few participants said they had been targets or witnesses of physical aggression, such as being pushed and shoved during demonstrations and anti-abortion pickets. (U) |  |
| Illustration | “Once, in front of an Italian hospital, there was a prayer group against abortion. These people pushed us. It was a bit unexpected; a minute before they were praying and immediately after they started shoving us.” ^(p.8)^ |  |
| Finding | In addition to the explicit aggressive behaviors noted, some participants declared they had experienced social disapproval. They had been targets of subtle and hidden negative actions, including disappointment, teasing, and temporary and/or permanent social distancing. (U) |  |
| Illustration | “I worked as a teacher at a high school for a year and shared my ideas (on abortion and my activism) with a co-worker. It became evident to me that my positionalities did not stimulate her interest in learning more about me, and she became increasingly distant. Furthermore, I have been the target of taunting in the past [for my opinion and positionalities].” ^(p.8)^ |  |
| Finding | In particular, participants asserted that most women who publicly defend the right to abort are often negatively considered as unemotional, unable to be a mother, misandric, lesbian, unnatural or crazy as well as career-oriented. Some also declared they were perceived as selfish and so oriented toward satisfying their own needs that they supported people who chose to give up their reproductive capacity. (U) |  |
| Illustration | “[…] There is a strange gaze against us … there is a judgment even on our couple’s relationships […] as women who affirm the right to choose and to have “the final say” [on reproductive choice]; we are considered women who neglect the other component of the couple […]. The other stereotype is about the quality of our maternal competence […]. Therefore, we don’t correspond to that stereotype of woman that society proposes to us.” ^(p.9)^  “[People think] we are women focused on our career or other forms of personal fulfillment. We are women who have lost the sense of family.“ ^(p.9)^ |  |
| Finding | On the one hand, they indicated that they are perceived as immoral and, in particular, accomplices in a murder and/or murderers, especially by those people with a right-wing political orientation, who are religious, or shared anti-abortion ideologies. (U) |  |
| Illustration | “[…] Those who have a right-wing political orientation or a religious vision do not support the voluntary interruption of pregnancy and think abortion is the murder of a child. I think that they can see us as monsters who want to kill children […].” ^(p.9)^ |  |
| Finding | On the other hand, they are often perceived as disreputable or promiscuous women who have had uncontrolled sexual relations and do not care about the consequences. (U) |  |
| Illustration | “We were all whores in the past, too […] Those who were against [abortion] obviously called us whores because we clearly encouraged women to be free to interrupt their pregnancy […]. With this common thinking of the time, having the right to abortion meant being free to be whores. There was this mentality and there still is” ^(p.9)^ |  |
| Finding | Furthermore, participants felt that activists are perceived to be politically constructed in a negative way. (U) |  |
| Illustration | “There are many people who look at us badly […]. They tell us “you are exaggerating [..] The Italian law is the best in the world”. They also add, “take it easy, laugh”. There is also counter-information from the Ministry of Health. Considering the last year, it seems that everything is going very well in abortion […]. From the institutional data it appears that there are no problems. People tell us “we are in 2018, we are not in the Middle Ages … Don’t worry.”” ^(p.10)^ |  |
| Finding | The participants also believed they were associated with non-human traits and compared to witches, priestesses of evil, and Satan. (U) |  |
| Illustration | “[The pro-choice activists] are seen as Satan. […] There is just the feeling that people perceive them as a bugbear, a devil. Women who choose to have an abortion are seen as sinners and activists as witches, the antichrist. There is this medieval perception of women.” ^(p.10)^ |  |
| Finding | They were regarded as a point of reference on abortion and able to encourage other individuals to take a stance on the termination of pregnancy. (U) |  |
| Illustration | “You become the person to ask for help when you need it […] you become a reference point for reproductive health.” ^(p.10)^ |  |
| Finding | Although the majority of the participants said they had been stigmatized and felt a negative gaze on themselves as a result of their activism, they were opposed to this depreciating view of themselves as a result of their abortion positionalities and their role in pro-choice movements, affirming their political and social value. (U) |  |
| Illustration | “Indeed, the activists described themselves as tenacious and proudly combative women who are determined, courageous, and enthusiastic in fighting for abortion rights with engagement” ^(p.11)^ |  |
| Finding | They stated that, despite their discomfort at times as a result of various obstacles, such as the actions of no-choice movements and the presence of abortion stigma, they do not give up on their goals, do not get discouraged or afraid. (U) |  |
| Illustration | “I think that activists are women who are not afraid to be stigmatized by the society […]. They are so convinced they are doing something important for all women that … I don’t know how to say … they don’t feel scared. I feel they are sometimes worried about how things are going about abortion, but they are … we are not afraid, we are worried.” ^(p.11)^ |  |
| Finding | One participant in particular linked being a nuisance to a strong sense of justice, which drives her to persevere even in the face of the most difficult challenges, such as combating abortion stigma. (U) |  |
| Illustration | “We are occasionally a nuisance […]. I believe I am [we are] a person with a sense of justice, in need of a little justice, and because of that I’m [we’re] a nuisance.” ^(p.11)^ |  |
| Finding | From a personal point of view, speaking openly allowed participants to proudly own a part of their identity and retain an internal sense of coherence, which helped them fight stigma internalization. (U) |  |
| Illustration | “I speak openly about it [activism] even if I know there is a prejudice. My ideas have always been quite clear […]. […] I have never been repressed. I have never done any kind of censorship and I prefer to talk because I think it [activism] is a part of me, an added value of my person” ^(p.12)^ |  |
| Finding | Instead, from a political point of view, the chance of freely speaking enabled them to deconstruct hostile speeches and attitudes toward abortion and people who are directly or indirectly involved in it. (U) |  |
| Illustration | “[…] I never hide. Speaking openly, the person in front of me can “turn his nose up” but can also benefit from my opinion.” ^(p.12)^ |  |
| Finding | In most cases, cisgender female pro-choice activists declared that abortion was the symbol of people’s self-determination and freedom, thus highlighting that making decisions for themselves was a way to claim their own needs and an opportunity to protect their psychological health and avoid unsustainable choices. (U) |  |
| Illustration | “I speak above all of the right to choose whether or not to be mothers, of the fact that motherhood is not destiny. Who is not ready, who does not have this in their life plan, who does not feel to be a mother, why should it be at any cost? Because society expects it from you? Because your parents want to be grandparents or because your partner insists? If you do it because it is a duty, because you are persuaded, because your parents force you to do so, because you feel a sense of guilt or shame […] you will never be able to build a healthy educational relationship with your kids.” ^(p.12)^ |  |
| Finding | In their opinion, this could counteract the negative vision that portrays women who decide to abort as women who choose without thinking about it. (U) |  |
| Illustration | “I always say that a woman does not abort because she wants to profess a right or because she is happy, it is always the last resort! Because […] in my opinion a woman arrives [at the decision] with great pain and as a last resort. I always try to highlight this because it is a great pain” ^(p.13)^ |  |
| Finding | Specifically, she declared that it was imperative to convey positive abortion experiences and raise awareness that abortion pain and distress are often merely the outcome of internalizing moral mistreatment. (U) |  |
| Illustration | “Abortion is frequently seen and described as one of the most traumatic experiences a woman can have … I don’t think so. There are women for whom it is so; […] then there are testimonies that tell of their own experience of abortion not as a negative experience that has marked them for life or traumatic. So also, showing different narratives on these issues is crucial.” ^(p.13)^ |  |
| Finding | According to them, selective self-censorship was useful to avoid contemptuous and judgmental glances and social conflicts, especially with people with firm negative attitudes about abortion. (U) |  |
| Illustration | “[I silenced my activism] especially at university. When I wrote my thesis in pharmacy, I attended a lab for a year. I hid it from my professor and tutor … they didn’t know that I was committed as an activist in different issues such as elective abortion, violence against women, and so on. … I still have a bit of fear in …I do not know how my activism can be considered. I felt that my activism would be seen as a negative thing … I’m not sure, but this was my gut feeling and, therefore, for this reason, I did not speak about it.” ^(p.13)^ |  |
| **Study: Mosley.** [33] **Sexuality‑based Stigma and Inclusion Among Southern Protestant Religious Leaders** | | |
| Finding | Similarly, despite clear examples of abortion stigma, there were also examples of supportive and affirming views on abortion and sexual and reproductive health, generally. (U) |  |
| Illustration | "My personal views on abortion? Hmm…That’s a decision that the individual and God should make." ^(p.1524)^ |  |
| **Study: Sisson et al.** [29]**“The stakes are so high”: interviews with progressive journalists reporting on abortion** | | |
| Finding | A frequent hurdle was the need to educate editors on abortion as both a medical procedure and political issue, which they did not need to do when reporting on other topics: (U) |  |
| Illustration | “It was very clear the editor thought… abortion required something akin to major surgery, like a c-section…There are a lot of assumptions that they bring to editing that are not born out in what we know about abortion.” ^(p.397)^  “I find myself explaining what I would hope a news editor would understand about healthcare, how these issues are related to one another… I have observed that my [abortion] pitches are more dismissed than others, and I'm not the only one that has made that observation.” ^(p.397)^ |  |
| Finding | Half of participants (n= 16) shared that they found it hard to identify new sources around abortion, whether they were seeking out providers, patients, or researchers: (U) |  |
| Illustration | "The biggest trouble with reporting on abortion is, as far as I'm concerned, the defensiveness of people at abortion clinics. [Clients] don't want to talk about it, [and] the doctors are very preoccupied with their work." ^(p.398)^  “One of the things that I have found most difficult over the years is finding academics who can talk about this… who are not also advocates of either side. There are a few, but not a lot. That's always been my biggest hurdle. In health care there's a bazillion academics. They're constantly bombarding me. That's so not the case in abortion” ^(p.398)^ |  |
| Finding | Most participants reported experiencing harassment as a result of abortion reporting; 24 of 31 participants had faced some form of it. (U) |  |
| Illustration | “Antis [anti-abortion advocates] tweeted out my home address. So that was an issue for me as a writer and it did have a chilling effect… It made me really terrified.” ^(p.398)^ |  |
| **Study: Smith *et al.*** [39] **Social Norms and Stigma Regarding Unintended Pregnancy and Pregnancy Decisions: A Qualitative Study of Young Women in Alabama** | | |
| Finding | Compared with parenting, abortion was also perceived as far less visible in participants’ communities. (U) |  |
| Illustration | “White participants were more likely to report that they did not know anyone who had an abortion, but speculated that abortion may happen “more often than people realize,” as a white FG participant who had 2 pregnancies reflects: “There’s probably more women that have had [an abortion], but it’s something they might be ashamed of and don’t tell anyone.” ^(p.10)^ |  |
| Finding | For most participants, the choice of abortion was only viewed as acceptable in “real trying circumstances,” which encompassed rape, drug abuse, severe mental illness, and homelessness. Abortion was also deemed permissible when a woman’s life is in danger or when fetal anomalies are detected, as a multiracial interviewee with no pregnancies shared. (C) |  |
| Illustration | “[If] my kid is going to have [a] crippling deformity [that] I can get rid of it beforehand, that might be okay or you end up having two twins and like one of them is partially conjoined, you can remove the conjoined one before they are born. Or they can pick up Down Syndrome now so you can just quit the pregnancy.” ^(p.11)^ |  |
| Finding | Participants imagined community members would react negatively to finding out that a young woman had an abortion, as a black FG participant with no pregnancies stated: (U) |  |
| Illustration | “Well like I’m from deep in the south… They kind of view it as a bad thing like you’re killing your child. That’s why most people in my community do keep their children because they really don’t want to kill a baby.” ^(p.11)^ |  |
| Finding | Young women who choose abortion and those involved in provision of abortion services were perceived as irresponsible and selfish, weak, coldhearted, and immoral, as a white interviewee with no pregnancies shared (U) |  |
| Illustration | “I think it’s horrible. I just wish they never created abortion clinics—the word abortion…I wish it never existed. I think it’s ridiculous. I think it’s all selfish.” ^(p.11)^ |  |
| Finding | Many of the white participants described interactions with crisis pregnancy centers, which provided them with free diapers, cribs, bottles, and “mommy money” for attending “classes and [watching] 15 minute videos.” A white FG participant who had one pregnancy describes the message delivered by one such organization during a visit to her high school: (U) |  |
| Illustration | “They try to get every girl to think that abortion is horrible, and if you’re going to have the baby…put it up for adoption if you don’t want it. ‘Do not, do not have an abortion… Abortion is horrible, you’ll get sick…’” ^(p.11)^ |  |
| Finding | Multiple participants shared stories of friends and community members who kept their abortions secret. (U) |  |
| Illustration | They voiced suspicions that women within their reference network who reported having a miscarriage really had “hidden abortion[s].” ^(p.11)^  “I know plenty of people that have had abortions that don’t even tell their doctors… They’re ashamed of it. They go [to abortion clinics] with a hood over their face … They know it’s wrong, but they think that’s what’s best for them or the child that they already have… They just can’t face the reality of going ahead and having another baby.” ^(p.11)^ |  |
