# Supplementary material for: Abortion stigma amongst the public in high-income countries: a mixed-method systematic review
Source: Sex Reprod Health Matters. 2026 Feb 9;33(1):2622203. doi: 10.1080/26410397.2026.2622203 (PMC13097179; doi:10.1080/26410397.2026.2622203)
Supplement: Supplementary Table 4. Abortion stigma definitions. [file ZRHM_A_2622203_SM4885.docx]

Supplementary Table 4. Abortion stigma definitions

| Citation | Cockrill and Nack (2013) | Hanschmidt et al.  (2016) | Kumar, Hessini and Mitchell (2009) | Letourneau (2016) | Martin et al. (2014) | Millar (2020) | Norris et al. (2011) | Ratcliffe et al. (2023) | Smith et al. (2016) | Only generic stigma  definition | Not  explicitly stated |
| --- | --- | --- | --- | --- | --- | --- | --- | --- | --- | --- | --- |
| Quantitative Studies | | | | | | | | | | | |
| Bommaraju et al. (2016) |  |  | 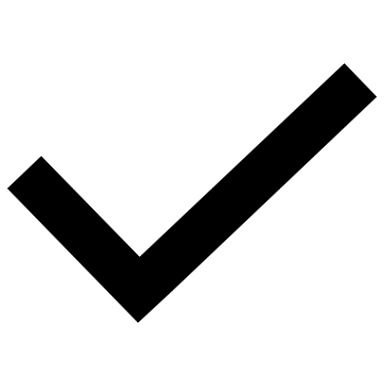 |  |  |  | 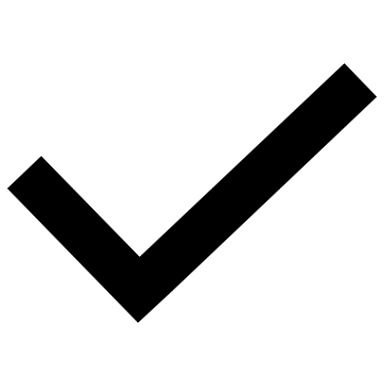 |  |  |  |  |
| Cutler et al. (2021) |  |  | 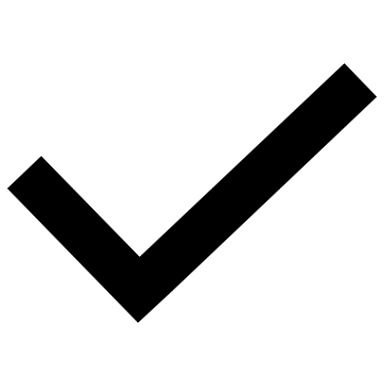 |  | 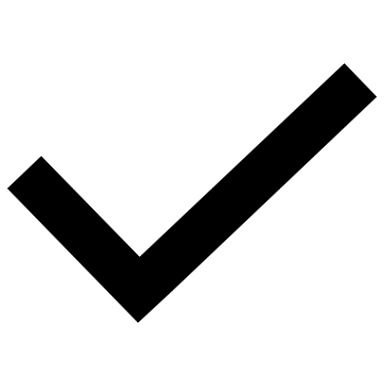 |  |  |  |  |  |  |
| Cutler et al. (2022) |  |  | 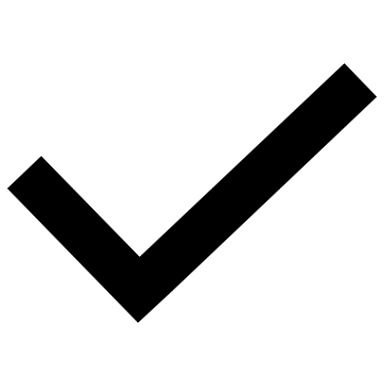 |  | 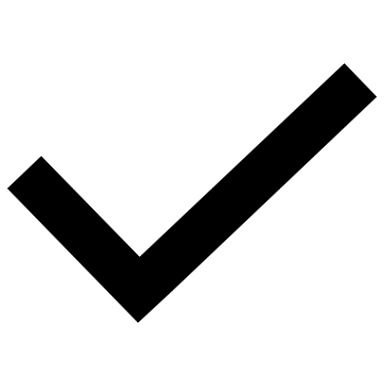 |  |  |  |  |  |  |
| Hanschmidt et al. (2020) |  |  | 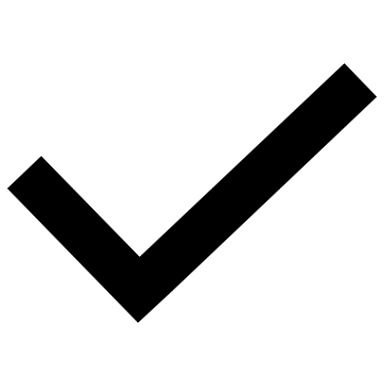 |  |  |  | 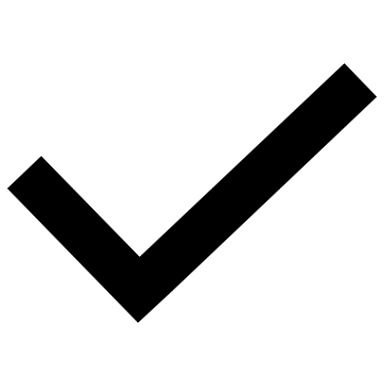 |  |  |  |  |
| Patev et al. (2019) |  | 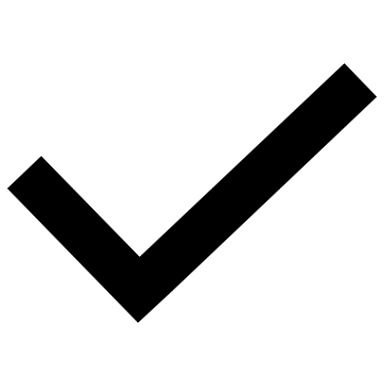 |  |  |  |  |  |  |  |  |  |
| Rice et al. (2017) |  |  |  |  |  |  |  |  | 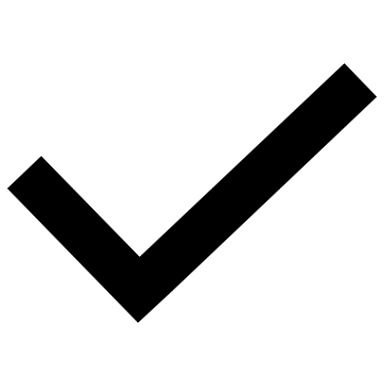 |  |  |
| Stowers et al. (2023) | 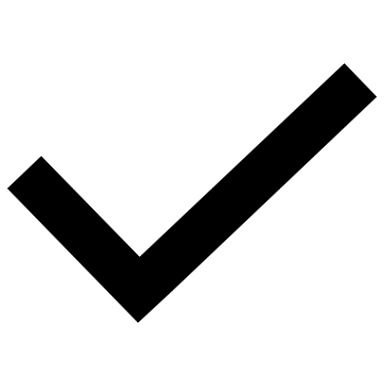 |  | 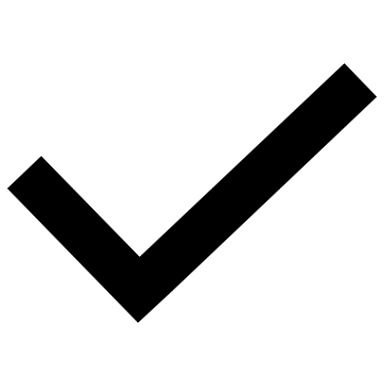 |  |  |  |  |  |  |  |  |
| Qualitative Studies | | | | | | | | | | | |
| Baker et al. (2023) |  |  | 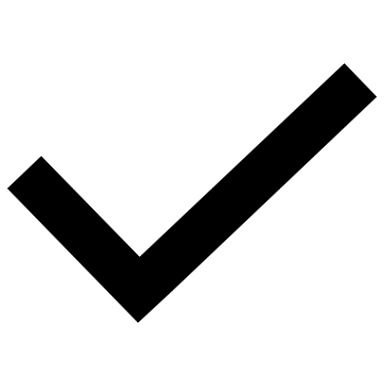 |  |  | 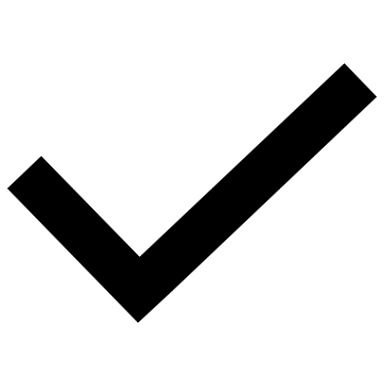 |  |  |  |  |  |
| Bloomer et al. (2024) | 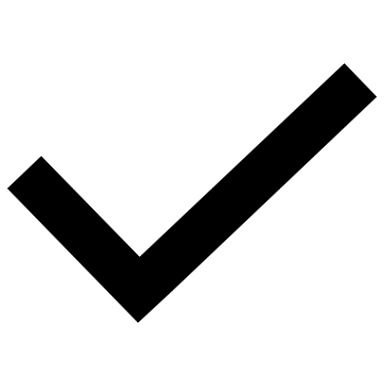 |  | 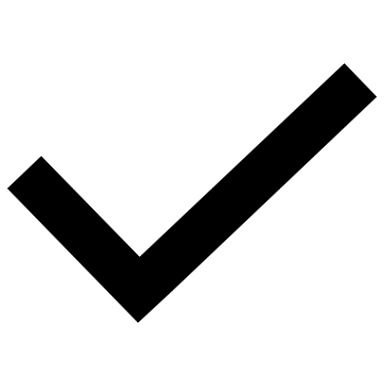 |  |  |  |  | 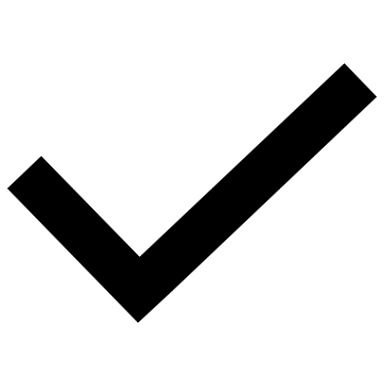 |  |  |  |
| Baird & Millar (2019) |  |  | 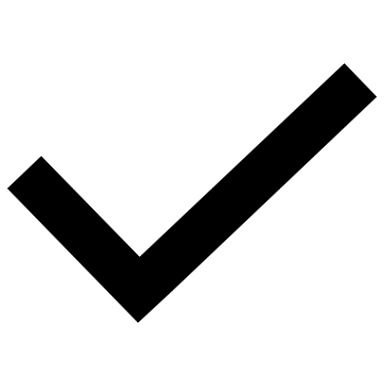 |  |  |  |  |  |  |  |  |
| Dianat et al. (2020) |  |  |  |  |  |  |  |  |  |  | 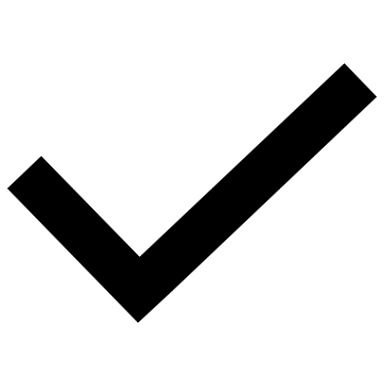 |
| Dozier et al. (2020) |  |  |  |  |  |  |  |  |  |  | 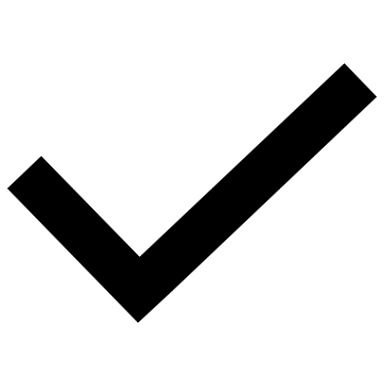 |
| Duerksen & Lawson (2017) |  |  |  |  |  |  |  |  |  |  | 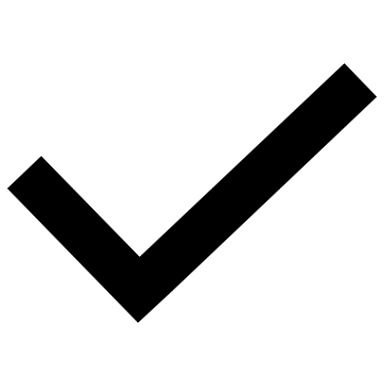 |
| Duerksen & Lawson (2018) |  |  | 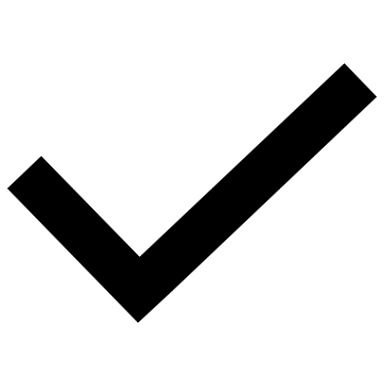 |  |  |  |  |  |  |  |  |
| Evans & O'Brien (2015) |  |  | 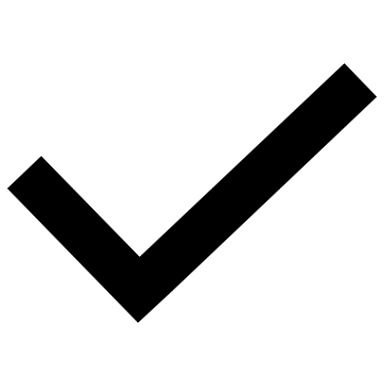 |  |  |  |  |  |  |  |  |
| Giovannelli et al. (2023) | 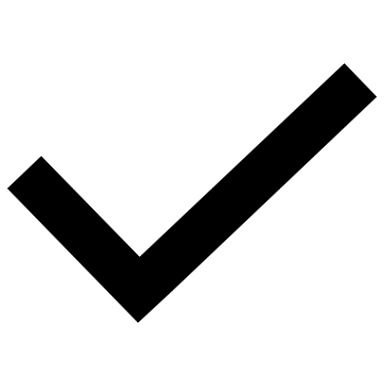 |  | 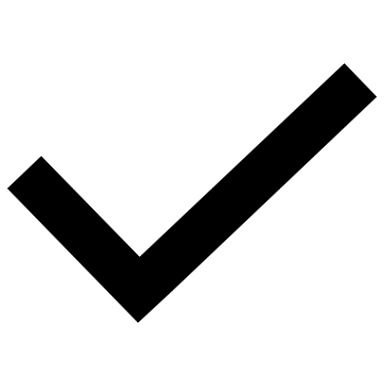 | 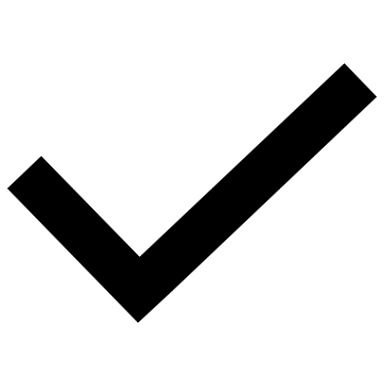 |  |  |  |  |  |  |  |
| Mosley et al. (2020) |  |  |  |  |  |  | 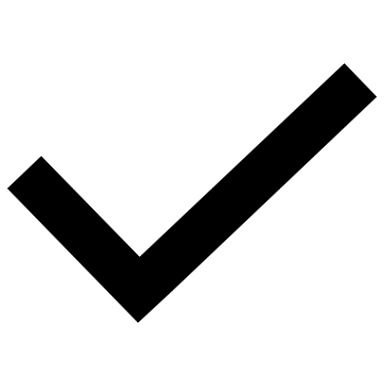 |  |  |  |  |
| Sisson et al. (2017) |  |  |  |  |  |  |  |  |  |  | 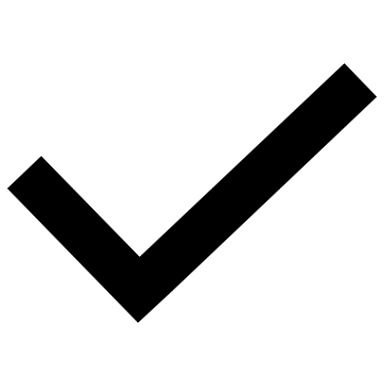 |
| Smith et al. (2016) |  |  |  |  |  |  |  |  |  | 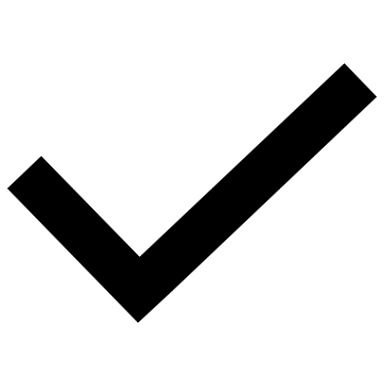 |  |
