# Supplementary material for: Abortion stigma amongst the public in high-income countries: a mixed-method systematic review
Source: Sex Reprod Health Matters. 2026 Feb 9;33(1):2622203. doi: 10.1080/26410397.2026.2622203 (PMC13097179; doi:10.1080/26410397.2026.2622203)
Supplement: Supplementary Table 5: Overview and Categorization of Quantitative Instruments. [file ZRHM_A_2622203_SM4884.docx]

Supplementary Table 5: Overview and Categorization of Quantitative Instruments

| Scale | Subscales and Items | Category | Studies |
| --- | --- | --- | --- |
| ASP  Abortion Stigma Perception | 1. It’s hard for other pregnant women to be around women who have had an abortion 2. Women are held more responsible for abortion than men are. 3. Most people are uncomfortable around women when they learn about their abortion. 4. When people know that a woman has had a abortion, they assume she will be a bad mother. 5. In general, telling others about an abortion is a mistake. 6. Most women who have an abortion feel like less of a woman. 7. Some women fear they’ll be treated badly because of their abortion 8. When people know that a woman has had an abortion], they look for ﬂaws in her character. | Perceived Stigma | (Bommaraju et al., 2016) |
| Abortion Norms and Stigma Scale | **Conditional Acceptability Scale (4 items)**   1. Abortion is acceptable if the woman cannot take care of her child. 2. Abortion is acceptable if the woman does not have family support. 3. If the man involved in the pregnancy will not support you in having a baby, it’s okay to have an abortion. 4. If your life is really messed up, it’s better to have an abortion than to keep the baby. | Acceptability | (Rice et al., 2017) |
|  | **Attitudes Scale (8 items)**   1. Abortion is acceptable in any situation. 2. Women who have abortions are killing their own children. 3. Abortion should be the woman’s decision. 4. In your opinion, how irresponsible is a woman your age who has an abortion. 5. In your opinion, how mature is a woman your age who has an abortion. 6. In your opinion, how selfish is a woman your age who has an abortion. 7. In your opinion, how strong is a woman your age who has an abortion. 8. In your opinion, how cold/heartless is a woman your age who has an abortion. | Attitudes/Stereotypes | (Rice et al., 2017) |
|  | **Anticipated Reactions Scale (7 items)**  *If I got pregnant accidentally and decided to have an abortion, the people who matter most to me:*   1. Would be disappointed. 2. Would be happy. 3. Would be mad. 4. Would be surprised. 5. Would understand. 6. Would be supportive. 7. Would feel ashamed. | Anticipated Stigma | (Rice et al., 2017) |
|  | **Misperceptions Scale (2 items)**   1. Abortion is risky for women’s health. 2. Women who have multiple abortions may not be able to have a child later in life. | Misperceptions | (Rice et al., 2017) |
| SABAS  Stigmatizing Attitudes, Beliefs, and Actions Scale | **Negative stereotyping SABAS items (8 Items)**   1. A woman who has an abortion is committing a sin. 2. Once a woman has one abortion, she will make it a habit. 3. A woman who has had an abortion cannot be trusted. 4. A woman who has an abortion brings shame to her family. 5. The health of a woman who has an abortion is never as good as it was before the abortion. 6. A woman who has had an abortion might encourage other women to get abortions. 7. A woman who has an abortion is a bad mother. 8. A woman who has an abortion brings shame to her community. | Attitudes/Stereotypes | (Patev et al., 2019) |
|  | **Exclusion and discrimination SABAS items (7 Items)**   1. A woman who has had an abortion should be prohibited from going to religious services. 2. I would tease a woman who has had an abortion so that she will be ashamed about her decision. 3. I would try to disgrace a woman in my community if I found out she’d had an abortion. 4. A man should not marry a woman who has had an abortion because she may not be able to bear children. 5. I would stop being friends with someone if I found out that she had an abortion. 6. I would point my fingers at a woman who had an abortion so that other people would know what she has done. 7. A woman who has an abortion should be treated the same as everyone else. | Enacted Stigma | (Patev et al., 2019) |
|  | **Fear of contagion SABAS items (3 Items)**   1. A woman who has an abortion can make other people fall ill or get sick. 2. A woman who has an abortion should be isolated from other people in the community for at least 1 month after having an abortion. 3. If a man has sex with a woman who has had an abortion, he will become infected with a disease. | Misperceptions | (Patev et al., 2019) |
| CAAS – Community Abortion Attitudes Scale | 1. Women who have had abortions are bad people 2. Women who have abortions have done something wrong 3. Women who have abortions should feel badly about themselves 4. If a friend of mine had an abortion, I would not judge her 5. I could support a woman who had an abortion even if I didn't agree with her decision 6. Abortion should be legal and available   Do you think abortion should be legal in all cases-illegal in all cases | Attitudes/Stereotypes | (Cutler et al., 2021, 2022) |
| REES –Experiences and Events Scale | 1. A woman who has had one abortion 2. A woman who becomes pregnant with triplets and chooses to selectively reduce to a single pregnancy 3. A woman who chooses to have an abortion because the fetus she is carrying has an abnormality 4. A women who has three abortions   A female doctor who performs abortions | Attitudes/Stereotypes | (Cutler et al., 2021, 2022) |

| CLASS  Community-Level Abortion Stigma Scale | **Stereotyping**   1. Women who have an abortion are stupid 2. Women who have an abortion are easywill sleep with anyone 3. Women who have an abortion are rejected 4. Men who allow their partners to have an abortion are rejected 5. Women who have an abortion deserve to be rejected 6. Women who have an abortion do not deserve to have a family 7. A man prefers to marry a woman who has never had an abortion 8. A decent woman would never have an abortion 9. An abortion causes a family shame 10. A woman is always at fault in an unwanted pregnancy 11. A woman without children is an incomplete woman | Attitudes/Stereotypes | (Stowers et al., 2023) |
| --- | --- | --- | --- |
|  | **Autonomy**   1. Women with children who choose to have an abortion do it to give the kids they already have a better life 2. A woman who has an abortion is doing what is right to not throw her life away 3. Women who have abortions because they feel unprepared to have children are responsible 4. It is ok that a woman has an abortion only because she does not want to have children at that moment | Acceptability | (Stowers et al., 2023) |
|  | **Religion**   1. Women who have abortions will be punished by God 2. Women who have abortions will receive divine punishment 3. Women who have abortions should go to Church to ask forgiveness for their actions 4. Resorting to more than one abortion is never justifiable | Attitudes/Stereotypes | (Stowers et al., 2023) |
|  | **Secrecy**   1. If you or your partner had an abortion, you would keep it a secret 2. Women who have an abortion should not tell anyone 3. Resorting to an abortion should be kept a secret as it is personal 4. If a woman had an abortion, she should not tell her future partners | Anticipated Stigma | (Cutler et al., 2021, 2022; Stowers et al., 2023) |
| Support for abortion access in case of | **Support for abortion access in case of…**   - … fetal health risk - … no more children - … woman’s health risk - … socio-economic restrictions - … rape - … single woman | Acceptability | (Hanschmidt et al., 2020) |
| Abortion legality attitudes | 1. Abortion is wrong, because everyone, even unborn babies have the right to life 2. Abortion should be illegal   It’s a woman’s constitutional right to choose whether or not to have an abortion | Acceptability | (Patev et al., 2019) |
| Support of abortion policies | **General support of abortion-related policies**   1. Please tell me whether or not you think it should be possible for a pregnant woman to obtain a legal abortion if she is married and does not want more children? 2. Please tell me whether or not you think it should be possible for a pregnant woman to obtain a legal abortion if she is not married? 3. Which of the following statements comes closest to your position on abortion? (“…legal and generally available” - “…banned and made illegal”) 4. In the United States, would you say there are… “…too many restrictions on abortion” - “…too few restrictions” 5. Below are some statements about the issue of abortion. Please indicate which one comes closest to your own view: “…personally believe having an abortion is acceptable and should be legal” … “…personally believe having an abortion is wrong and should be illegal” 6. The Supreme Court’s 1973 Roe v. Wade decision established a woman’s constitutional right to have an abortion. Which of the following statements comes closest to what you would like to see the Supreme Court do regarding its Roe v. Wade decision? “…should be overturned” … “…should not be over-turned” | Acceptability | (Cutler et al., 2021) |
|  | **Conditional support of abortion-related policies**   1. Please tell me whether or not you think it should be possible for a pregnant woman to obtain a legal abortion if she became pregnant as a result of rape? 2. Please tell me whether or not you think it should be possible for a pregnant woman to obtain a legal abortion if the woman’s own health is seriously endangered by the pregnancy? 3. Please tell me whether or not you think it should be possible for a pregnant woman to obtain a legal abortion if there is a strong chance of serious defect in the baby? | Acceptability | (Cutler et al., 2021) |
